# Optimization of Reduced Graphene Oxide/Titanium Dioxide-Coated Polyurethane Foams as Novel Floating Photocatalysts for Water Decontamination

**DOI:** 10.3390/molecules31152733

**Published:** 2026-08-06

**Authors:** Natalia Elia, Francesca De Rosa, Anna Dotti, Andrea Basso Peressut, Roberto Matarrese, Saverio Latorrata

**Affiliations:** 1 Department of Chemistry, Materials and Chemical Engineering “Giulio Natta”, Politecnico di Milano, Piazza Leonardo da Vinci 32, 20133 Milano, Italy; natalia.elia@polimi.it (N.E.); francesca.derosa@mail.polimi.it (F.D.R.); anna.dotti@polimi.it (A.D.); 2 Laboratory of Catalysis and Catalytic Processes, Department of Energy, Politecnico di Milano, Via La Masa 34, 20156 Milano, Italy; roberto.matarrese@polimi.it

**Keywords:** water decontamination, reduced graphene oxide, titanium dioxide, adsorption, photocatalysis, polyurethane foams, dip-coating, Rhodamine B, integrated photocatalytic adsorbents

## Abstract

Titanium dioxide (TiO_2_) photocatalysis is a promising sustainable solution for water decontamination; however, the industrial application of TiO_2_ is hindered by its wide band gap and high handling and recovery costs. This work proposes to overcome these issues by developing a composite coating of nanopowder TiO_2_ and reduced graphene oxide (rGO) applied onto commercial polyurethane (PU) foams by means of a simple, low-energy dip-coating process designed to enhance pollutant adsorption and photocatalytic activity. The rGO-TiO_2_ coating was optimized by exploring different surface pre-treatments of the PU foams, the deposition of rGO-TiO_2_ multilayers, and the variation in the rGO-TiO_2_ mass ratio (1:3, 1:4, and 1:5). The prepared materials were characterized by optical microscopy, SEM-EDX, DSC, and thermogravimetry, while the stability of the coating was preliminarily evaluated through ultrasonic tests. The water decontamination capability of the coated foams was investigated by adsorption and UV-Vis photodegradation tests using a 3 mg/L aqueous solution of Rhodamine B (RhB) as a model contaminant. The results demonstrated that the rGO-TiO_2_ 1:3-coated samples achieved complete RhB photodegradation within 90 min, with a pseudo-first-order kinetic constant of 0.0446 1/min. Moreover, pre-treating the foam with a 3 M NaOH solution improved coating adhesion onto the PU substrate while maintaining comparable decontamination efficiency.

## 1. Introduction

Each day, significant quantities of industrial, agricultural, and municipal waste are discharged into aquatic ecosystems, posing severe risks to both environmental and public health [1,2]. This continuous release of non-biodegradable toxic compounds (e.g., synthetic dyes, per- and polyfluoroalkyl substances (PFAS)) and pollutants (e.g., heavy metals, microplastics, industrial effluents) into water bodies contributes to the reduction in potable water availability [3,4]. Consequently, the development and implementation of effective strategies for wastewater treatment, recovery, and reuse have become increasingly urgent [1,2,5,6].

Among the available water decontamination techniques, adsorption is widely recognized as a promising approach due to its cost-effectiveness, operational simplicity, and high efficiency in the removal of metal ions, particularly when porous adsorbents are employed [7,8,9,10]. However, its practical application is often limited by low selectivity, slow kinetics, and difficulties associated with adsorbent regeneration and reuse [7,11,12,13,14]. In contrast, membrane separation enables rapid and efficient pollutant removal, even from complex mixtures that are difficult to treat through conventional separation processes [15,16]. However, membrane fouling, high energy consumption, and limited long-term durability continue to hinder its widespread implementation [17,18].

A particularly promising alternative is represented by photocatalysis, which has attracted increasing attention owing to its high efficiency against a wide range of pollutants, minimal operational costs, and environmental sustainability [19]. This process relies on the light-induced excitation of photocatalytic materials: upon irradiation, electrons and holes are generated and separated into the conduction and valence bands, respectively. These charge carriers react with water and oxygen to produce highly reactive radical species, such as hydroxyl (OH) and superoxide (O_2_^•−^) radicals, capable of oxidizing and degrading organic molecules into harmless or less harmful compounds, including CO_2_ and H_2_O [20]. Furthermore, the possibility of exploiting solar irradiation as an activation source enhances its appeal, enabling contaminant degradation under mild operating conditions [21,22,23]. Among the various photocatalytic materials investigated to date, titanium dioxide (TiO_2_) is one of the most extensively studied and widely applied owing to its chemical stability, low toxicity, and large-scale industrial availability [24,25]. Despite these advantages, its practical application is limited by the rapid recombination of photogenerated electron-hole pairs and by its relatively wide band gap (~3.2 eV), which restricts activation primarily under UV irradiation [22,26,27]. Moreover, TiO_2_ is commonly employed as nanoparticles, which tend to agglomerate, reducing the number of accessible active sites and thereby the overall photocatalytic efficiency [28,29]. The recovery and reuse of dispersed nanoparticles after treatment also remain challenging, further limiting their large-scale implementation [24,26].

Combining TiO_2_ with graphene oxide (GO), particularly in its reduced form (rGO), helps overcome these limitations [30]. Several studies have reported that GO-TiO_2_ and rGO-TiO_2_ composites exhibit a reduced effective band gap [31,32,33,34,35], thereby extending photocatalytic activity into the visible-light region. In addition, rGO acts as an efficient electron acceptor, facilitating charge transfer from TiO_2_ and suppressing electron-hole recombination, which ultimately improves photocatalytic performance [36,37,38]. Its functional groups also promote the formation of rGO-TiO_2_ architectures, where TiO_2_ nanoparticles are immobilized and uniformly dispersed, thereby minimizing nanoparticle aggregation [39,40,41,42,43]. Additionally, it has been demonstrated that, owing to its large specific surface area and reactive functional groups, the presence of rGO improves the decontamination performance of TiO_2_ by enhancing the adsorption of organic molecules [44,45] and metal ions [46,47]. As a result, the synergistic combination of rGO-assisted adsorption and TiO_2_-driven photodegradation enables rGO-TiO_2_ composites to act as integrated photocatalytic adsorbents, in which adsorption and photocatalysis occur simultaneously and cooperatively, leading to enhanced overall decontamination efficiency [44,45,48].

Despite these significant improvements, the practical implementation of TiO_2_/rGO composites in water treatment systems remains challenging because of issues related to material handling, recovery, and reuse [49,50,51,52]. Indeed, although suspended photocatalyst particles offer high specific surface areas and favorable mass transfer between pollutants in the liquid phase and the photocatalyst surface [53], they also present several drawbacks, including the difficult separation from treated water, particle agglomeration, and limited light penetration within the reaction medium [54]. Consequently, to overcome these limitations, photocatalytically active materials are frequently immobilized onto suitable support structures that provide mechanical stability and structural integrity while facilitating their recovery after treatment [50,55,56,57,58,59]. In particular, increasing attention has been devoted to the immobilization of visible-light-active semiconductor photocatalysts. For example, modified ZnO-based materials have been applied onto a variety of substrates, including glass, polymeric supports, and carbon-based materials, using techniques ranging from simple dip-coating procedures to more sophisticated approaches such as hydrothermal synthesis and electrospinning [60]. Miao et al. [61] immobilized a tailored BiOBr architecture onto a porous carbon-based aerogel and employed it for adsorption and visible-light-driven degradation of Rhodamine B, demonstrating the advantages associated with facile recovery and separation from treated water. Similarly, Barrocas et al. [62,63] immobilized two perovskite-type manganese oxides onto glass substrates by radio-frequency magnetron sputtering, obtaining visible-light-active photocatalysts for Rhodamine 6G decolorization. Notably, one of the materials retained its photocatalytic efficiency after repeated use and simple washing [62], highlighting the superior reusability and operational stability achievable through catalyst immobilization.

In this context, TiO_2_-based nanocomposite coatings have been applied to a wide range of substrates, including 2D supports such as natural and synthetic polymeric membranes [64,65], flat rigid substrates such as glass [66], and flexible cotton or polyamide textiles [67], as well as 3D supports like optical fibers [68] and melamine sponges [69]. Among these alternatives, 3D porous supports are particularly attractive because their interconnected porosity offers a high surface-area-to-volume ratio and low flow resistance [44,70]. Open-cell foams coated with photocatalytic materials offer extensive interfacial areas and improved light penetration, thereby promoting efficient pollutant transport and degradation. Consequently, they can outperform conventional 2D supports and achieve photocatalytic performance comparable to those of nanoparticle suspensions [71,72,73,74], while avoiding the recovery issues typically associated with dispersed materials. Polyurethane (PU) foams are especially suitable as support materials owing to their low cost, scalability, low density, high specific surface area, and remarkable adsorption capacity [75,76,77,78]. In addition, pristine PU exhibits notable affinity toward hydrophobic organic pollutants such as oils and petroleum derivatives [79]. Their porous architecture facilitates TiO_2_ immobilization and enhances photocatalytic performance [70]. TiO_2_/PU composites exhibit good mechanical stability, maintaining activity over multiple reuse cycles [75,78], and show no significant ecotoxicity toward aquatic organisms [77].

In a previous study [80], the self-assembly properties of rGO were successfully exploited to deposit mixed rGO-TiO_2_ coatings onto commercial floating PU foams through a simple and low-energy dip-coating and air-blowing process. While promising results were obtained, some critical aspects remained unresolved, particularly with regard to the partial detachment of the coating material. In addition, preliminary observations indicated that increasing the TiO_2_ content could further enhance the photocatalytic performance of the composite.

For this reason, the present work investigates different strategies aimed at improving coating stability, adhesion, and the amount of immobilized active material. To this end, PU foams were pre-treated with a 3 M NaOH solution as an alternative to other commonly reported surface modification methods [81,82,83]. In addition, different rGO-TiO_2_ ratios and multilayer coating configurations were also explored. The resulting materials were characterized by optical microscopy, SEM-EDX, DSC, and thermogravimetry. Preliminary ultrasonic tests were conducted to assess coating stability. Subsequently, the water decontamination performance of the coated foams was assessed through adsorption and photocatalytic degradation tests using an aqueous Rhodamine B (RhB) solution (3 mg/L) under UV-Vis irradiation. The kinetics of the process and the possible release of RhB from the foams into water after decontamination were also investigated.

## 2. Results and Discussion

### 2.1. Foam Coating Deposition and Characterization

The complete characterization of the coating components (rGO and TiO_2_), together with that of virgin PU as well as rGO- and rGO-TiO_2_ 1:3-coated foams, has been reported in previous studies [40,80,84]. In the present work, PU, rGO-, and rGO-TiO_2_ 1:3-coated foams are employed as reference systems to assess the effects of NaOH pre-treatment, of the deposition of multilayer coatings, and of the increase in TiO_2_ loading.

#### 2.1.1. 3 M NaOH Pre-Treatment

The NaOH pre-treatment has been proposed in the literature as a way of inducing surface modifications in the PU foam through alkaline hydrolysis [85]. In particular, it has been suggested that the interaction between PU and alkaline solutions may increase surface roughness and generate new active sites, potentially enhancing the interaction between the substrate and the coating components [86,87,88,89]. However, as reported in Table S1, no significant variation in foam weight was observed after the pre-treatment step, indicating a low hydrolysis degree. Therefore, it can be hypothesized that the pre-treatment primarily affects the most external region of the starting material.

**Figure 1:**
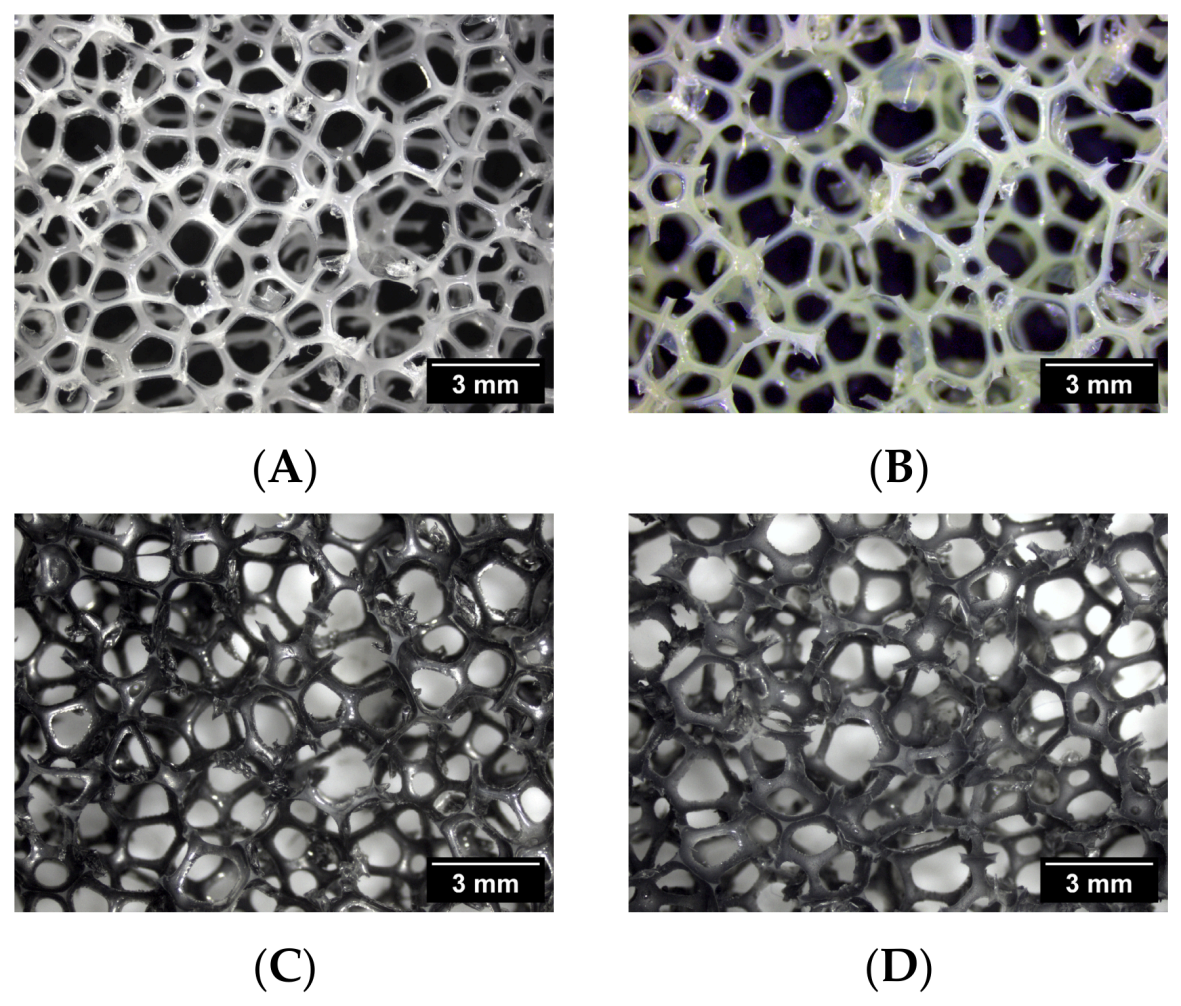
Optical microscopy images of virgin PU foam (**A**), bare pre-treated PU foam (**B**), pre-treated PU foams coated with rGO (**C**), and rGO-TiO_2_ 1:3 (**D**) coatings at 10× magnification. The images of pre-treated samples refer to the US-300-60-R0 sample, selected as the best-performing case. As all samples showed comparable visual features, only representative images are reported.

Virgin PU foams have a matte white appearance and a porous structure characterized by irregular pores (Figure 1A) with sizes ranging from several hundred micrometers to a few millimeters. Following the coating and washing steps, as previously shown in [80], the color of the foam samples shifted to black due to the presence of rGO. The incorporation of TiO_2_ led to coatings with qualitatively higher opacity and lower reflectivity compared to those composed solely of rGO. In all cases, the use of compressed air proved effective in removing excess coating while preserving the porous structure of the foam [80]. The alkaline pre-treatment with 3 M NaOH induced a partial yellowing of the uncoated PU substrate (Figure 1B), which was most likely associated with the formation and deposition of reaction products on the foam surface and was largely removed after washing. Despite this temporary effect, both rGO- and rGO-TiO_2_ 1:3-coated samples (Figure 1C,D) displayed a homogeneous coating distribution.

**Figure 2:**
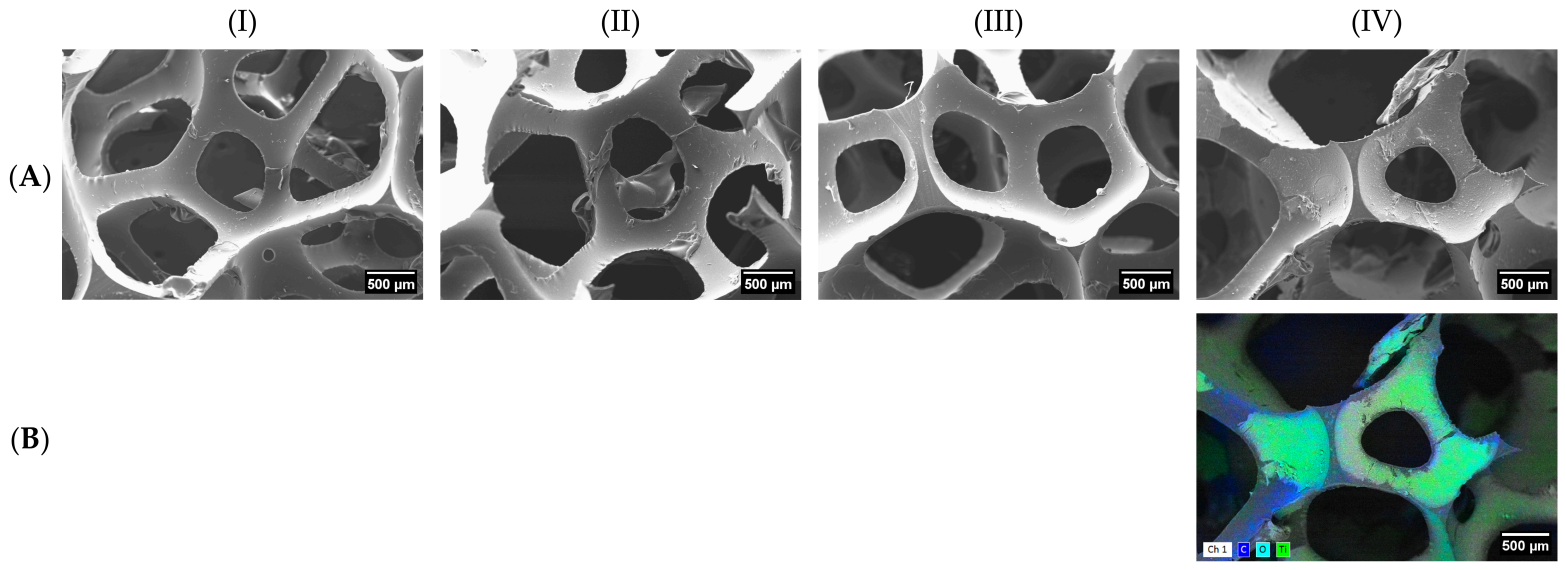
SEM images acquired using secondary electrons (**A**) of virgin PU (I), bare 3 M NaOH pre-treated PU (II), rGO-coated 3 M NaOH pre-treated foam (III), and rGO-TiO_2_ 1:3-coated 3 M NaOH pre-treated foam (IV). EDX elemental map (**B**) of the 3 M NaOH pre-treated foam coated with the rGO-TiO_2_ 1:3 formulation (IV). The images of pre-treated foams refer to the US-300-60-R0 sample, selected as the best-performing case. As all samples showed comparable visual features, only representative images are reported.

SEM-EDX analyses provided further insight into both the foam morphology and the elemental composition of the applied coatings. In the case of bare PU foams pre-treated with NaOH (Figure 2, II), no significant differences were observed compared to untreated PU (Figure 2, I), although slight irregularities at the foam edges could be noticed. This observation, coupled with the low hydrolysis degree (Table S1) and the slight reduction in oxygen content after the pre-treatment evidenced by the EDS semi-quantitative results (Table S3), suggests that the alkaline treatment did not induce substantial changes in either the surface morphology or surface composition of the foam. Interestingly, Somani et al. [89] reported weight losses below 1% for PU samples treated for 1 h in a 10% NaOH solution at 60 °C. This value increased with treatment time, ranging from approximately 2% for 2 h of treatment to 6.5% for 10 h. These observations indicate that PU hydrolysis proceeds relatively slowly since the interaction between the aqueous NaOH solution and the pristine PU surface may be limited due to hydrophilic-hydrophobic interactions. Therefore, under the treatment conditions adopted in this work, namely room temperature and short treatment duration, the pre-treatment may have primarily acted as a cleaning step, removing residual impurities left from the PU foam manufacturing process and thereby promoting a mild surface activation of the outermost foam layers. Such an effect may have contributed to a more favorable interaction between the substrate and the deposited coating, without causing pronounced surface etching or extensive surface modification.

The application of the coating resulted in the formation of uniform layers (Figure 2, III and IV, Figure S1), with Ti homogeneously distributed over the foam surface (Figure 2B, IV).

The effectiveness of the coating process was evaluated by quantifying the amount of deposited material and assessing its mechanical stability through ultrasonic stability tests. Table 1 and Table 2 summarize the data obtained for both rGO- and rGO-TiO_2_ 1:3-coated foams pre-treated with 3 M NaOH, respectively. In the case of rGO-coated samples (Table 1), the pre-treatment led, in almost all cases, to an increase in the amount of deposited material, ranging approximately from 3% up to about 40% compared to the untreated sample. The best results were obtained in the case of US-300-60-R0 (300 mL, 1 h, US) and ST-100-60-R0 (100 mL, 1 h, stirring) samples, with coating masses of 9.2 mg and 9.7 mg, respectively, compared to 6.9 mg for the untreated foam. No clear correlation was observed between the number of reuse cycles of the NaOH solution and the amount of deposited material, as evidenced by tests US-100-30-R0 to US-100-30-R4. This behavior is consistent with the stability of the NaOH solution after reuse, as confirmed by the titration results (Table S2), which indicate a moderate decrease in concentration after five cycles. Conversely, a dependence on pre-treatment duration was observed for the stirred samples (ST-100-60-R0, ST-100-120-R0, and ST-100-240-R0), where increasing the treatment time resulted in a decrease in the amount of deposited material. As reported in the literature [90], longer NaOH treatments might initially enhance coating adhesion through increased surface roughness; however, excessively prolonged treatments might lead to an over-etching of the foam morphology, resulting in smoother surfaces and, consequently, reduced coating deposition efficiency. Concerning rGO-TiO_2_ 1:3-coated samples (Table 2), the highest deposited mass was recorded for tests US-100-30-R0 (100 mL, 30 min, US) and ST-100-120-R0 (100 mL, 120 min, stirring). No clear correlation was observed between the amount of deposited material and both the number of solution reuse cycles and the pre-treatment duration. However, in line with the results discussed above, the pre-treatment generally seems to promote a higher amount of deposited coating, with an increase ranging approximately from 2% up to 46% compared to the untreated case.

Regarding the stability tests, in the case of rGO-coated foams (Table 1), no evident trends were observed, with CLus (i.e., the percentage of coating loss after the ultrasonic test) values both lower and higher than that obtained without the pre-treatment. However, the best result in terms of CL_US_ was registered for US-300-60-R0 (300 mL, 1 h, US). Instead, regarding rGO-TiO_2_ 1:3-coated samples (Table 2), all NaOH pre-treatments resulted in a decrease in CL_US_, with a markedly better result obtained with the US-300-60-R0 procedure. Hence, having provided the best coating stability for both coating types, while also improving the amount of deposited material compared to untreated foams, the US-300-60-R0 treatment was selected for further investigations. Representative SEM images of coated foams before and after stability tests are reported in Figure S3. These micrographs show that the coating remains uniformly distributed over the foam surface after testing, with no evidence of coating detachment, thereby qualitatively highlighting the beneficial effect of the pre-treatment on coating stability.

Although the adhesion mechanism was not directly investigated, the adhesion of dip-coated rGO-based coatings to polymeric substrates is generally attributed to a combination of weak intermolecular interactions, such as van der Waals forces, electrostatic interactions [91,92] and, where suitable surface functionalities are available, hydrogen bonding [93] between urethane groups of the polyurethane substrate and the residual oxygen-containing functional groups of rGO [94,95,96]. Therefore, it can be hypothesized that the mild surface activation induced by the pre-treatment may have promoted a more favorable interaction between the polyurethane surface and the rGO sheets. Even though limited, this effect may have facilitated the establishment of interfacial interactions involving the residual oxygenated groups of rGO, thereby enhancing coating adhesion and retention on the substrate.

**Figure 3:**
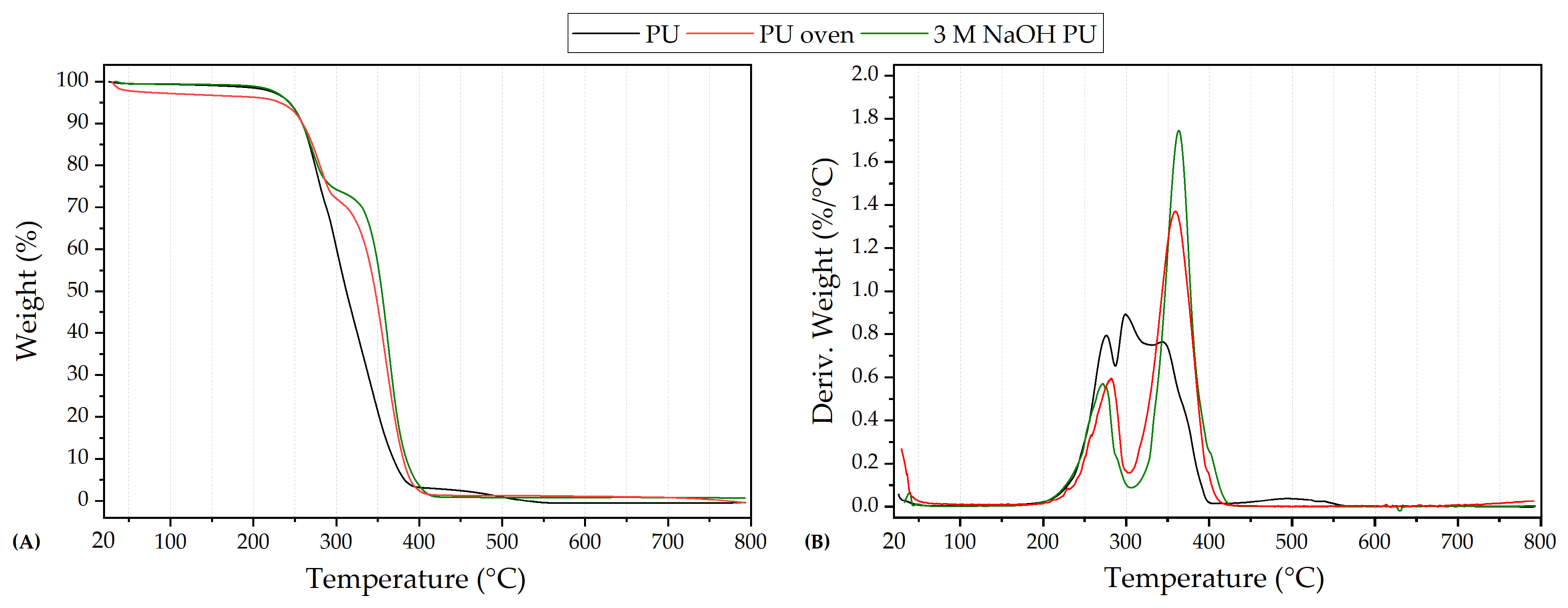
TGA (**A**) and DTG (**B**) plots of bare untreated and pre-treated PU foams.

**Figure 4:**
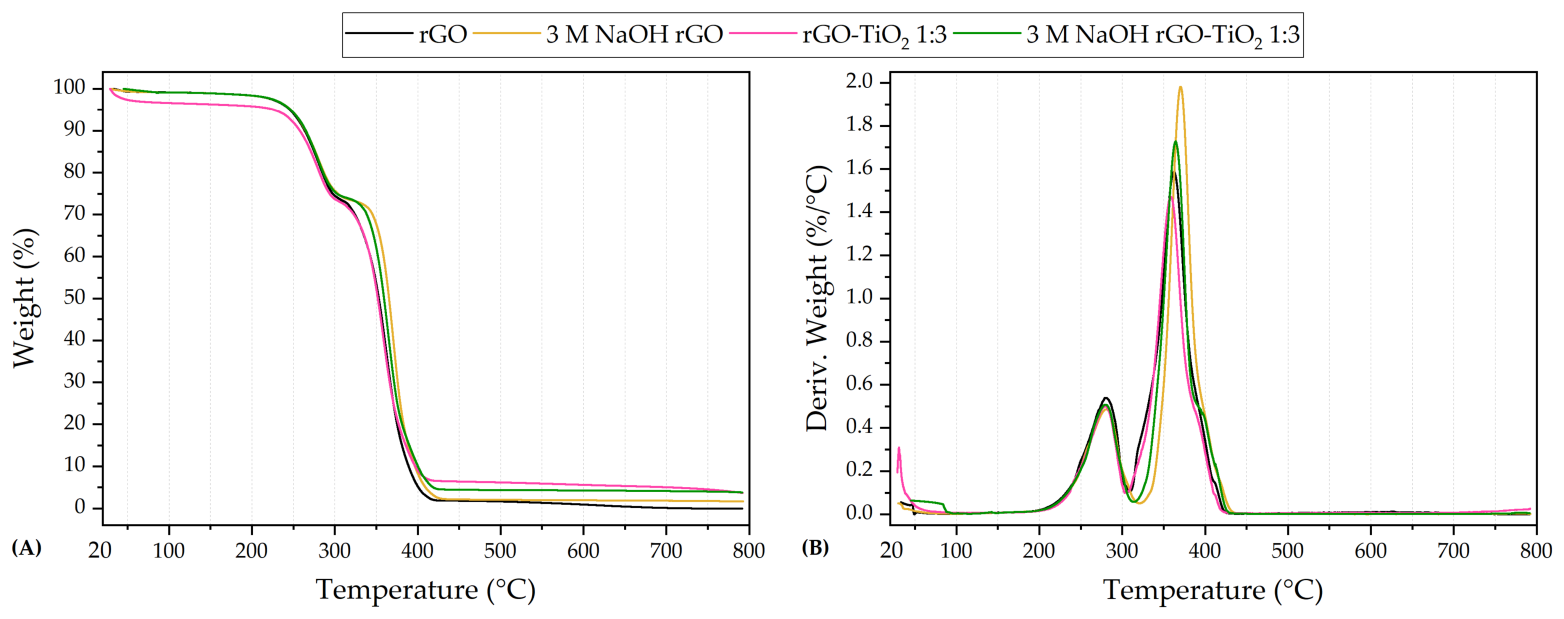
TGA (**A**) and DTG (**B**) plots of rGO- and rGO-TiO_2_ 1:3-coated untreated and pre-treated PU foams.

The thermal behavior of bare and coated foams was analyzed by TGA (Figure 3 and Figure 4). The thermal characterization of virgin PU, rGO- and rGO-TiO_2_ 1:3-coated foams, as well as the possible effects of coating deposition on the PU properties, were previously reported in [80]. Briefly, virgin PU undergoes a complex thermal decomposition process between 200 °C and 500 °C, associated with the degradation of hard segments (250–350 °C), followed by that of soft segments (350–500 °C). Due to the low coating loading, the thermal contributions of rGO are not clearly distinguishable in the TGA curves; however, the presence of TiO_2_ is reflected by the higher residual mass at 800 °C. The differences in TGA and DTG curves observed for bare and coated foams were attributed to the 40 °C oven-drying step applied during sample preparation, rather than to the presence of the coating [80].

Figure 3 and Figure 4 show the thermal profiles of 3 M NaOH pre-treated uncoated and coated foams. Since the alkaline pre-treatment involves not only immersion in a 3 M NaOH solution but also a drying step, an uncoated foam subjected only to heating at 40 °C for 1 h (“PU oven” in Figure 3 and Figure 4) was included as a reference sample. This allows the effect of the alkaline treatment to be distinguished from any changes associated with the drying step alone. Figure 3A shows that untreated (“PU oven”) and treated PU (“3 M NaOH PU”) foams, subjected to the same thermal treatment, exhibit similar mass-loss profiles. The main thermal phenomenon occurs in the same temperature range for both materials, with only minor differences. As reported in Table S4, the 3 M NaOH foam shows a slight shift in the initial mass loss toward lower temperatures (the T_onset_ decreases from approximately 250 °C for PU oven to 242 °C for 3 M NaOH PU) and a slightly higher residual mass at 800 °C (0% for PU oven and 0.6% for the pre-treated material). This suggests that the pre-treatment does not significantly affect the overall thermal stability of the PU foam. A similar trend can be observed in the DTG curves (Figure 3B): besides a minor shift in the position of the two degradation peaks (i.e., the peak associated with hard segments shifted towards lower temperatures, from 283 °C to 271 °C, whereas that related to soft segments towards higher temperatures, from 359 °C to 363 °C), the treated sample exhibits a higher and sharper peak, suggesting a faster and more uniform decomposition of the soft segments. Despite these subtle differences, the overall degradation profile remains unchanged, confirming that the pre-treatment has only a limited impact on the thermal stability of the PU foams. This effect is also consistently observed across all coated samples (Figure 4), further supporting the idea that even the presence of the coating does not influence the thermal properties of the support material.

These findings are further supported by the analysis of the third heating ramp of the DSC curve (Figure S2A). As in the case of TGA/DTG, the thermal behavior of virgin PU, rGO-, and rGO-TiO_2_ 1:3-coated foams has already been discussed in [80]. In summary, virgin PU exhibits an initial thermal event (30–70 °C) that is absent in coated samples, suggesting that the oven-drying step applied during sample preparation may be responsible for a permanent modification of the PU structure. A second thermal transition, observed at approximately 114 °C, corresponds to the glass transition temperature (T_g_) and is associated with the mobility of the hard segments of the PU structure. Consistent with the TGA results, comparison of the DSC thermograms of bare and coated foams confirms that the presence of the coating does not significantly affect the thermal behavior of the PU substrate [80].

In the case of pre-treated foams (Figure S2A), all samples exhibit a broad transition centered around 114 °C, which can be associated with the glass transition temperature (T_g_) of the PU foam. Thus, the close similarity in both the shape of the curves and the corresponding transition temperatures confirms that neither the alkaline pre-treatment nor the presence of the coating significantly affects the thermal behavior of the substrate.

#### 2.1.2. Multilayer Coatings

**Figure 5:**
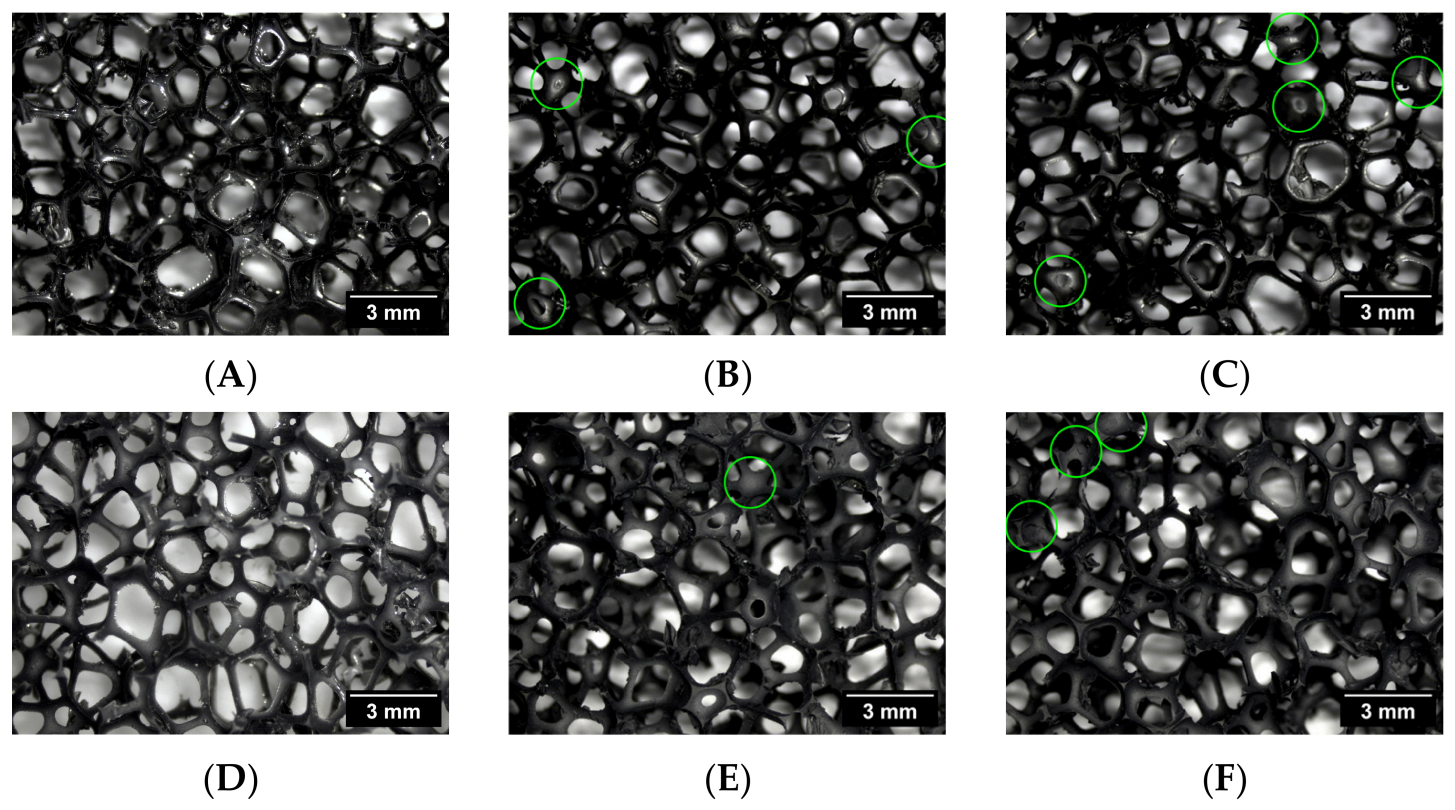
Optical microscopy images of PU foams coated with rGO (one layer (**A**), two layers (**B**), and three layers (**C**)) and rGO-TiO_2_ 1:3 (one layer (**D**), two layers (**E**), and three layers (**F**)) at 10× magnification.

A multilayer coating strategy was also selected to try to increase the amount of active material deposited on the foam. As reported in the literature, it is known that the formation of multilayer films enhances the interaction between TiO_2_ and rGO, favoring the formation of Ti–O–C bonds, which enhance charge separation and the generation of reactive species [97,98]. Moreover, it was hypothesized that the layered structure may contribute to increased coating porosity and surface area, potentially improving pollutant adsorption and light interaction, thus enhancing photocatalytic efficiency. Optical microscopy images (Figure 5) show that the application of two or three successive layers of the same coating resulted in a progressive partial clogging of the foam pores (e.g., green circles in Figure 5), although preserving the open-cell structure of the PU foam.

**Figure 6:**
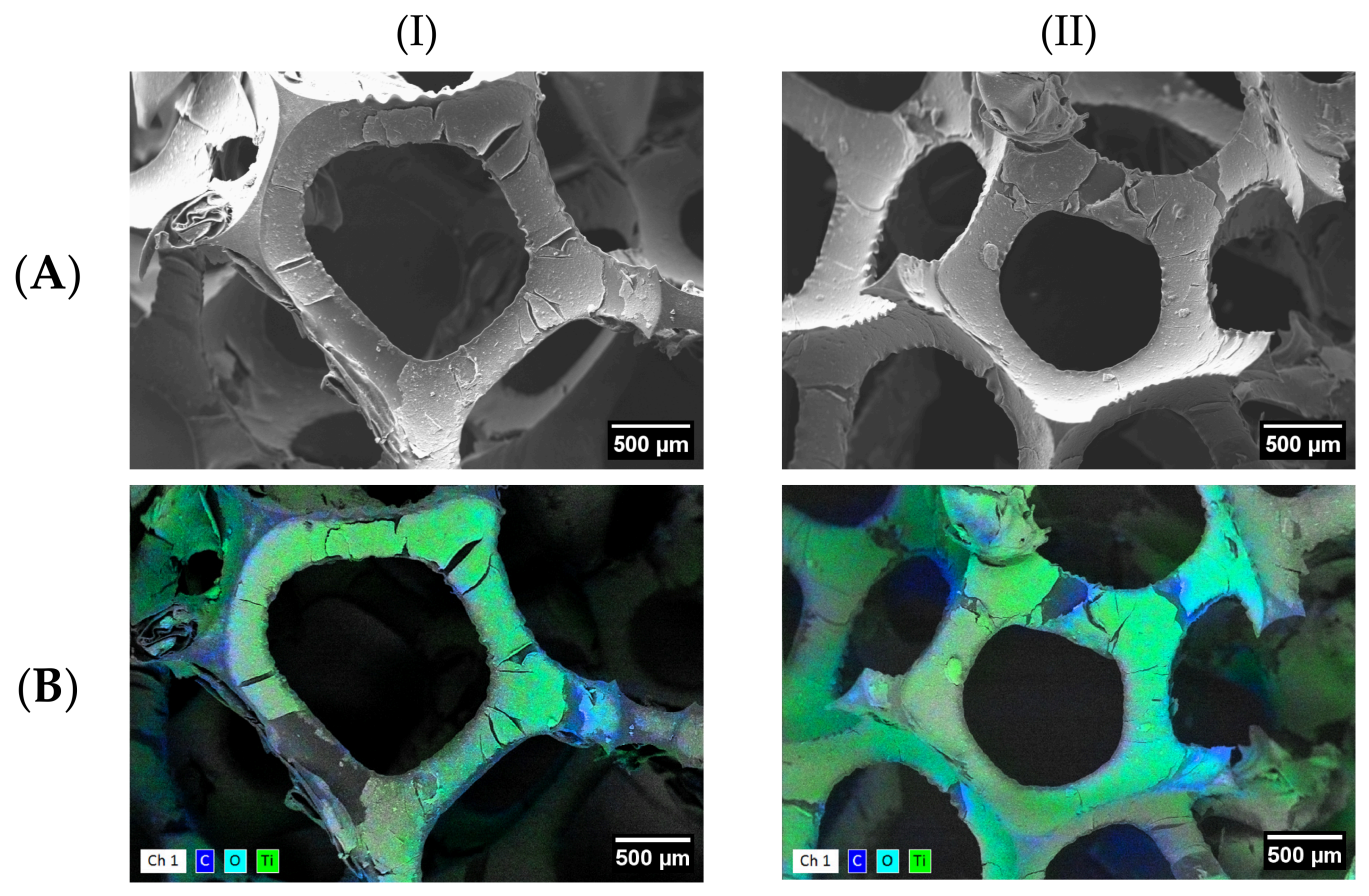
SEM images acquired using secondary electrons (**A**) and EDX elemental maps (**B**) of PU foams coated with 2 layers (I) and 3 layers (II) of rGO-TiO_2_ 1:3.

SEM-EDX images (Figure 6) reveal highly irregular surfaces, characterized by a discontinuous coating distribution, with areas where the coating is absent and others where it is present but exhibits fractures. The presence of two and three distinct coating layers is also clearly visible in Figure S4.

Considering the amount of material deposition (Table 3), the highest values were observed during the first coating step (~80 mg for rGO and ~90 mg for rGO-TiO_2_ 1:3, before immersion in deionized water). Subsequent depositions resulted in smaller mass increments, with lower contributions from the second layer (27.3 mg and 28.4 mg for rGO; 62.3 mg and 65.0 mg for rGO-TiO_2_ 1:3) and from the third layer (26.2 mg for rGO, and 56.6 mg for rGO-TiO_2_ 1:3). However, the higher deposited mass is associated with increased detachment of fragments during the washing cycle, indicating limited inter-layer adhesion and suggesting that interactions between adjacent layers are based on physical interactions, rather than on chemical bonding. Stability tests (Table 3) further confirm a progressive decrease in coating integrity as the number of deposited layers increases. In particular, rGO-coated samples exhibited higher stability compared to their rGO-TiO_2_ counterparts, likely due to the intrinsic self-assembly properties of GO [99]. On the other hand, coatings containing TiO_2_ showed greater mass loss, suggesting that TiO_2_ may disrupt the structural continuity of rGO, particularly in multilayer configurations. Despite these adhesion challenges, the application of a second and third layer resulted in an increase in the amount of deposited material compared to a single coating, thus achieving the desired goal of increasing the amount of deposited active material.

#### 2.1.3. Increase in TiO_2_ Loading

**Figure 7:**
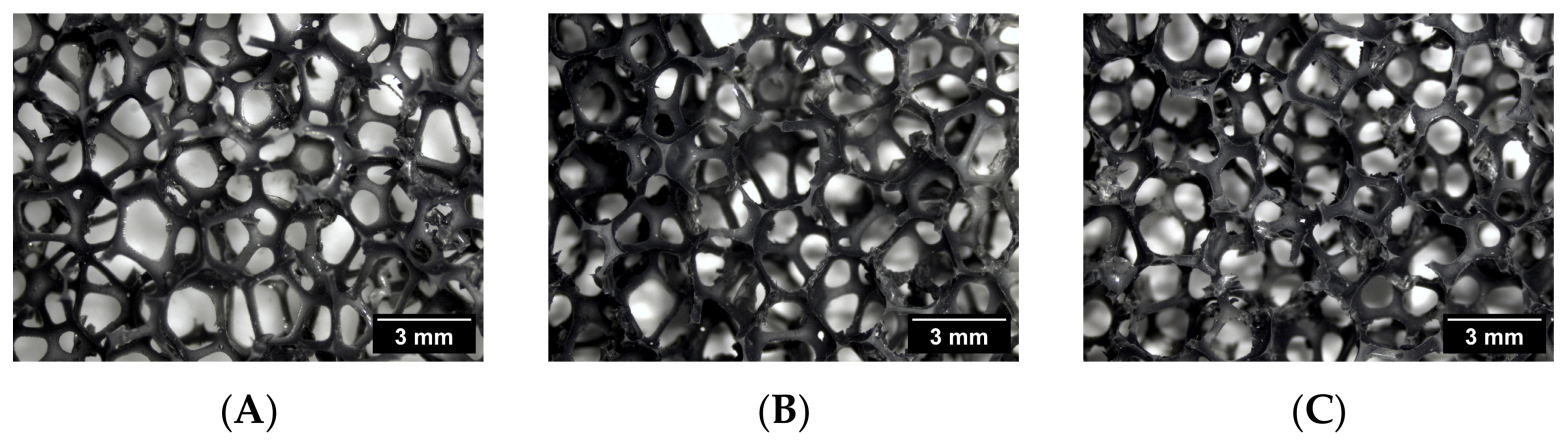
Optical microscopy images of PU foams coated with rGO-TiO_2_ 1:3 (**A**), rGO-TiO_2_ 1:4 (**B**), and rGO-TiO_2_ 1:5 (**C**) mass ratios at 10× magnification.

In a previous study by the authors [80], it was demonstrated that increasing the TiO_2_ content in the coating formulation leads to enhanced photocatalytic performance of the developed rGO-TiO_2_-coated foams, with the best performance (100% RhB decontamination in 90 min) obtained with a rGO:TiO_2_ mass ratio of 1:3. For this reason, rGO:TiO_2_ ratios of 1:4 and 1:5 were investigated in this work to verify whether the decontamination performance could be further enhanced. As shown in Figure 7, increasing the TiO_2_ content in a single layer (rGO:TiO_2_ ratios of 1:4 and 1:5) resulted in slightly higher opacity compared to the 1:3 formulation. Moreover, the presence of numerous white spots within the coating layer indicated the formation of more TiO_2_ clusters, suggesting that the increased TiO_2_ loading hindered its homogeneous dispersion.

**Figure 8:**
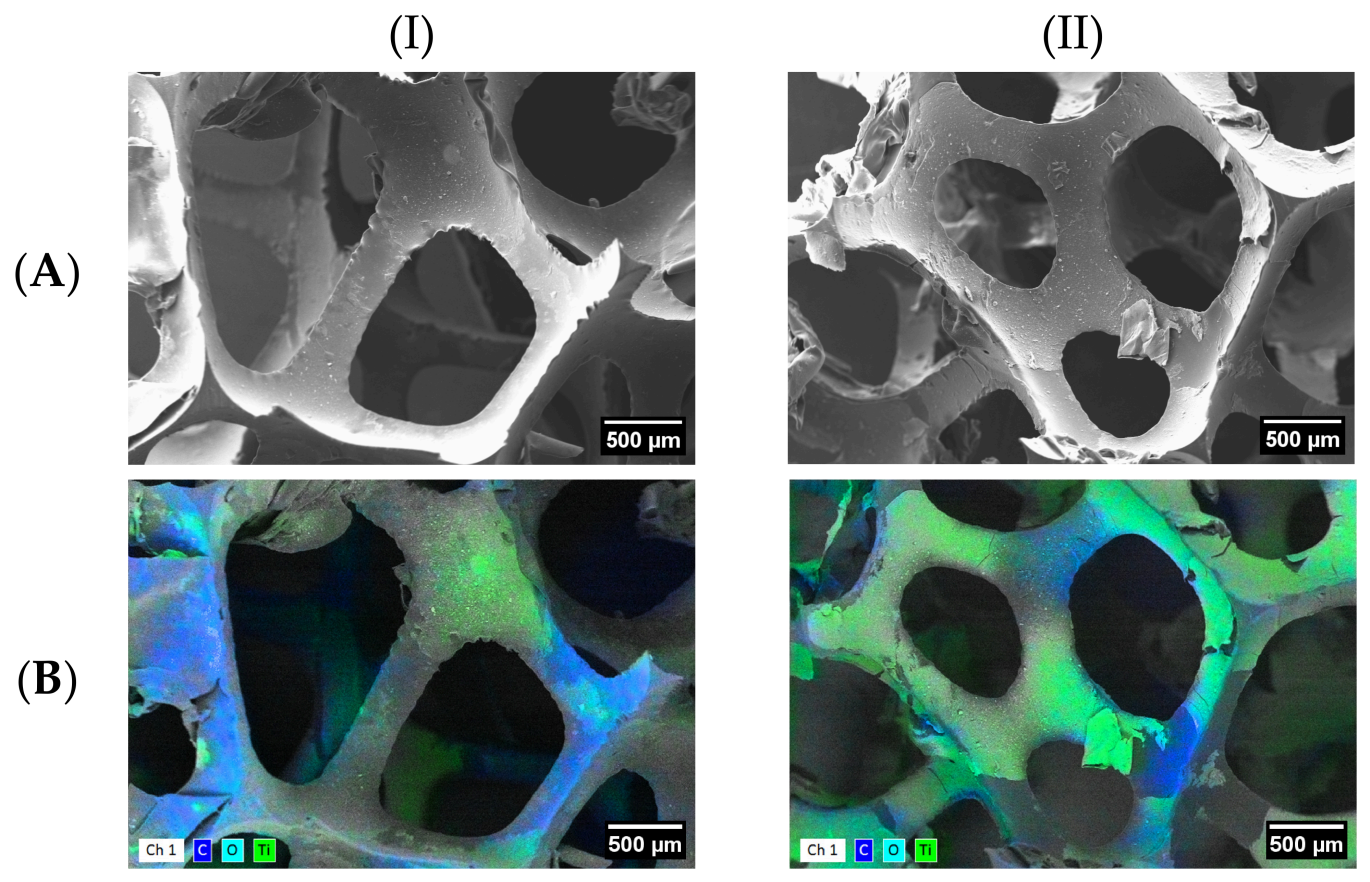
SEM images acquired using secondary electrons (**A**) and EDX elemental maps (**B**) of rGO-TiO_2_ 1:4- (I) and rGO-TiO_2_ 1:5- (II) coated PU foams.

With higher titania loadings within the formulation (Figure 8A), the surface appears highly inhomogeneous, both in terms of coating distribution and TiO_2_ distribution within the layer. In fact, TiO_2_ agglomerates are clearly visible in Figure S5. In particular, the high-magnification images (Figure S5 II to IV) reveal the presence of numerous TiO_2_ particles with a heterogeneous size distribution, while the number of aggregates progressively increases from the 1:3 to the 1:5 formulation. Furthermore, EDS elemental maps (Figure 8B) show a highly irregular Ti distribution, confirming the non-uniform nanopowder dispersion within the deposited coating.

Table 4 highlights a corresponding increase in the deposited coating mass as the TiO_2_ loading rises (38.5 mg for rGO-TiO_2_ 1:4 and 44.6 mg for rGO-TiO_2_ 1:5). However, when moving from rGO:TiO_2_ = 1:3 to rGO:TiO_2_ = 1:4, this increase is not proportional, resulting in a higher deposited mass than expected, whereas a less marked increase is observed upon further increasing the TiO_2_ content. This suggests that the deposited coating mass does not only depend on the higher mass contribution of TiO_2_, but also on other factors such as the reduced mechanical continuity of the coating at higher TiO_2_ loadings, which may lead to increased fragment detachment during the washing cycle. This increased material detachment was also observed in the stability test results (CLus = 44.2% for rGO-TiO_2_ 1:4 and CLus = 54.6% for rGO-TiO_2_ 1:5), indicating a possible progressive coating embrittlement as the TiO_2_ content increases.

**Figure 9:**
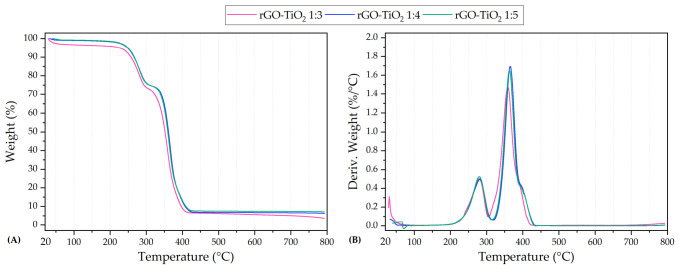
TGA (**A**) and DTG (**B**) plots of rGO-TiO_2_ 1:3-, 1:4-, and 1:5-coated PU foams.

In the case of rGO-TiO_2_ 1:4 and 1:5 foams, the TGA/DTG curves (Figure 9) exhibit very similar thermal degradation profiles, with no significant differences among the three formulations. As summarized in Table S5, all samples undergo degradation through the same two-step process and display comparable onset temperatures (around 247 °C for 1:3, around 247 °C for 1:4, and around 250 °C for 1:5), maximum degradation temperatures (T_max_ = 280–281 °C for the first degradation stage and 359–365 °C for the second one), and weight losses in each degradation step (23–24% and 68%, respectively). The only noticeable difference is related to the residual mass at high temperature, which rises with increasing TiO_2_ content (3.7% for 1:3, 6.3% for 1:4, and 7.1% for 1:5).

Figure S2B shows the DSC curves for rGO-TiO_2_ 1:4- and 1:5-coated samples. Also in this case, increasing the TiO_2_ content does not modify the DSC profiles or the T_g_, indicating that higher TiO_2_ loadings do not induce additional thermal events. Overall, these results support the conclusion that variations in the coating do not significantly affect the thermal behavior of the PU substrate.

### 2.2. Water Decontamination

The applicability of the coated foams for water treatment was evaluated using the samples obtained after the most promising 3 M NaOH pre-treatment, as well as those produced with multilayer coatings and using rGO-TiO_2_ mass ratios of 1:4 and 1:5. Their performance was compared with that of the reference samples, i.e., bare PU, rGO-, and rGO-TiO_2_ 1:3-coated foams.

**Figure 10:**
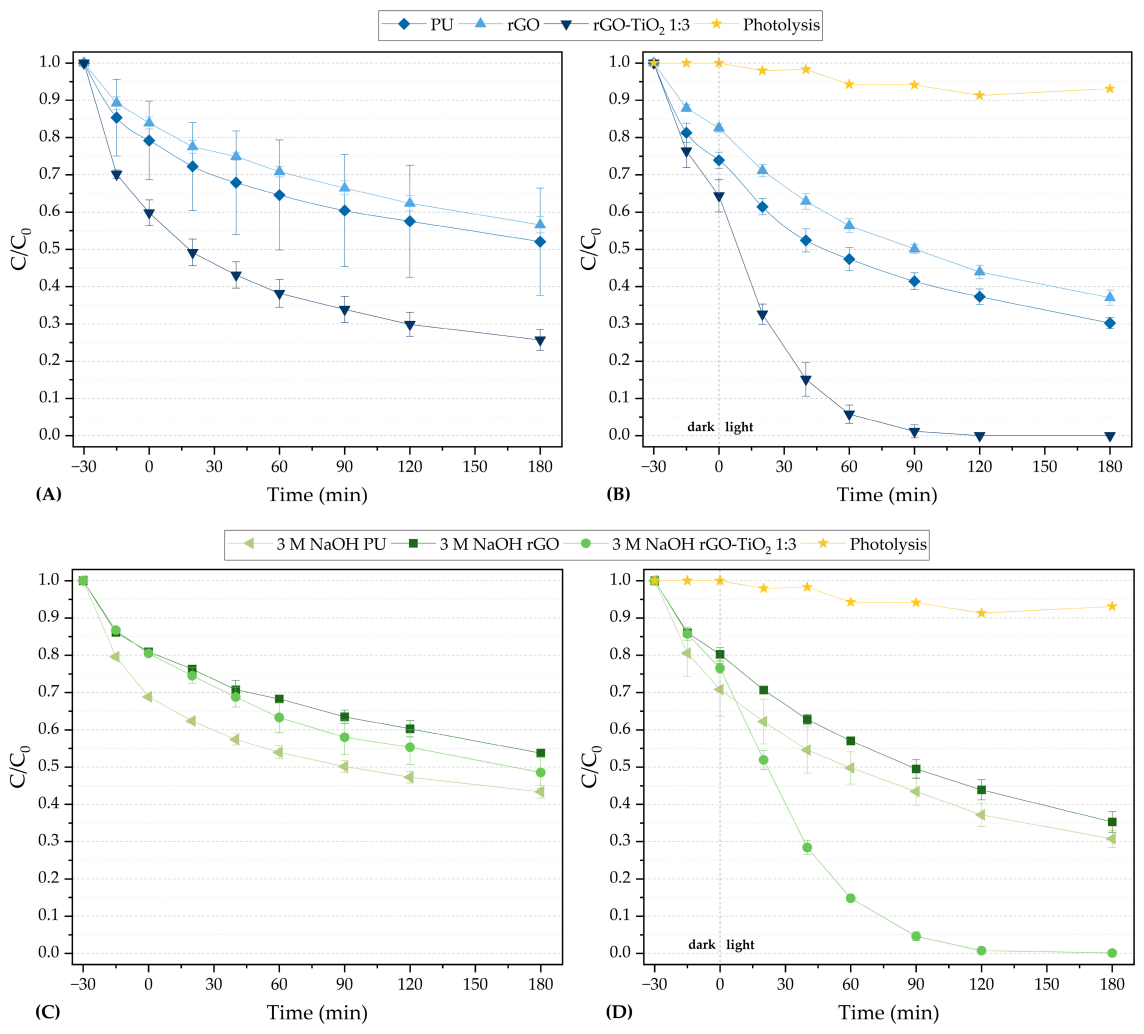
Normalized concentration of RhB solution during dark (**A**,**C**) and light test (**B**,**D**) with 3 M NaOH pre-treated samples (**bottom**) and untreated references (**top**).

Starting from 3 M NaOH pre-treated foams, Figure 10 summarizes the results from tests carried out both in the dark and under UV-Vis irradiation. In the dark (Figure 10A,C), the process relies exclusively on adsorption. Bare untreated PU exhibited a significant adsorption capacity, reaching a RhB removal of 48% (Figure 10A). The presence of a rGO coating led to a slight reduction in adsorption performance (43% removal), while the rGO-TiO_2_ 1:3 coating enhanced the removal efficiency, reaching 74% of RhB removal. In the case of pre-treated foams (Figure 10C), bare pre-treated PU showed a slightly higher average uptake (57%) than bare untreated PU. However, considering the experimental variability, no definitive conclusion can be drawn regarding the effect of the alkaline treatment on the interaction between PU foam and RhB. Instead, removal values of 46% and 51% were recorded for rGO- and rGO-TiO_2_ 1:3- coated pre-treated foams, respectively. Under UV-Vis irradiation (Figure 10B,D), rGO and bare PU samples exhibited a very similar trend, reaching RhB removal values of approximately 70% for virgin and pre-treated PU (Figure 10B), 63% for rGO and 65% for rGO treated with 3 M NaOH (Figure 10D). Considering photolysis as negligible, the improved performance of PU compared with that observed under dark conditions might be attributed to light-induced alterations of the foam surface morphology [80], while for rGO-coated foams, the enhancement in removal efficiency may be related to the occurrence of some photocatalytic phenomena [100]. In TiO_2_-containing coatings, the activation of the UV-Vis lamp at 0 min resulted in a change in the curve slope, suggesting a transition from pure adsorption (occurring during the initial 30 min in the dark) to a combined process in which adsorption coexists with photodegradation induced by TiO_2_ activation [101]. As a result, complete RhB removal was achieved in 90 min by untreated rGO-TiO_2_ 1:3 samples and in 120 min for the pre-treated counterpart (95.4% RhB removal in 90 min). Despite this lower photodegradation efficiency, 3 M NaOH pre-treated samples showed substantially improved coating stability (Table 5), thus preventing the release of coating fragments into the solution. This aspect is particularly relevant for the intended application. Indeed, the effectiveness of immobilized photocatalysts is not solely determined by their degradation efficiency, but also by their ability to preserve the active phase under operating conditions, thereby enabling material recovery and reuse. Coating detachment can progressively reduce the available photocatalytic surface and compromise the benefits associated with catalyst immobilization. The importance of these aspects has been highlighted in previous studies on immobilized TiO_2_-based materials [102,103,104,105,106,107,108,109]. For example, Giannakis et al. [103] reported that immobilized TiO_2_ films on silicon substrates maintained their photocatalytic performance over five consecutive degradation cycles without significant changes in the observed reaction rate, while SEM analyses before and after photocatalytic testing revealed no appreciable alterations in surface morphology, confirming the structural stability of the deposited photocatalytic layer. These findings emphasize that the long-term effectiveness of immobilized photocatalysts relies on their ability not only to maintain a stable photocatalytic performance but also to preserve coating integrity during operation. Therefore, the enhanced adhesion observed for the 3 M NaOH-treated foams suggests a potentially more durable photocatalytic system, in which a moderate reduction in short-term activity may be compensated by improved stability during operation.

**Figure 11:**
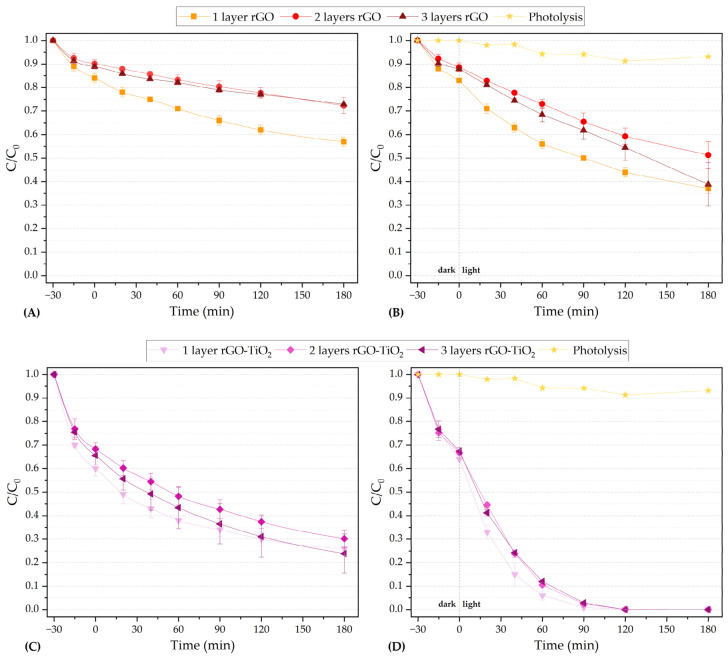
Normalized concentration of RhB solution during dark (**A**,**C**) and light (**B**,**D**) tests for multilayer rGO- (**top**) and rGO-TiO_2_- (**bottom**) coated samples.

The performance of multilayer-coated samples in RhB removal under dark conditions and UV-Vis irradiation was evaluated and compared with that of the corresponding single-layer coatings (Figure 11). Under dark conditions, increasing the number of rGO layers (Figure 11A) significantly reduced the removal efficiency (43% for one layer, 28% and 27% for two and three layers, respectively, after 210 min of immersion). In contrast, the presence of TiO_2_ mitigated this effect, leading to removal efficiencies of 70% for two layers and 76% for three layers, compared to 74% for the reference rGO-TiO_2_ 1:3 monolayer (Figure 11C). Thus, despite the greater amount of deposited material, additional layers did not enhance the adsorption performance. Under UV-Vis irradiation, multilayer rGO coatings (Figure 11B) showed higher RhB removal than in the dark (49% for two layers, 61% for three layers), but still inferior to that of the single-layer rGO reference sample (63%). In the case of rGO-TiO_2_ 1:3 coatings (Figure 11D), multilayer samples required slightly longer times to achieve complete RhB removal: 120 min for both two and three layers, compared to 90 min for the single-layer coating. After 90 min, two- and three-layer coatings achieved 98% and 97% removal, respectively. Overall, despite the higher amount of deposited material, increasing the number of layers did not result in measurable improvements in either adsorption performance or global RhB removal under irradiation. This may be attributed to a combination of factors, including reduced accessibility of active sites associated with partially clogged pores and increased coating thickness, which can hinder contaminant diffusion toward the underlying layers, thereby limiting adsorption. Moreover, shielding effects from the upper layers may cause light attenuation, thereby restricting the photoactivation of the inner layers.

**Figure 12:**
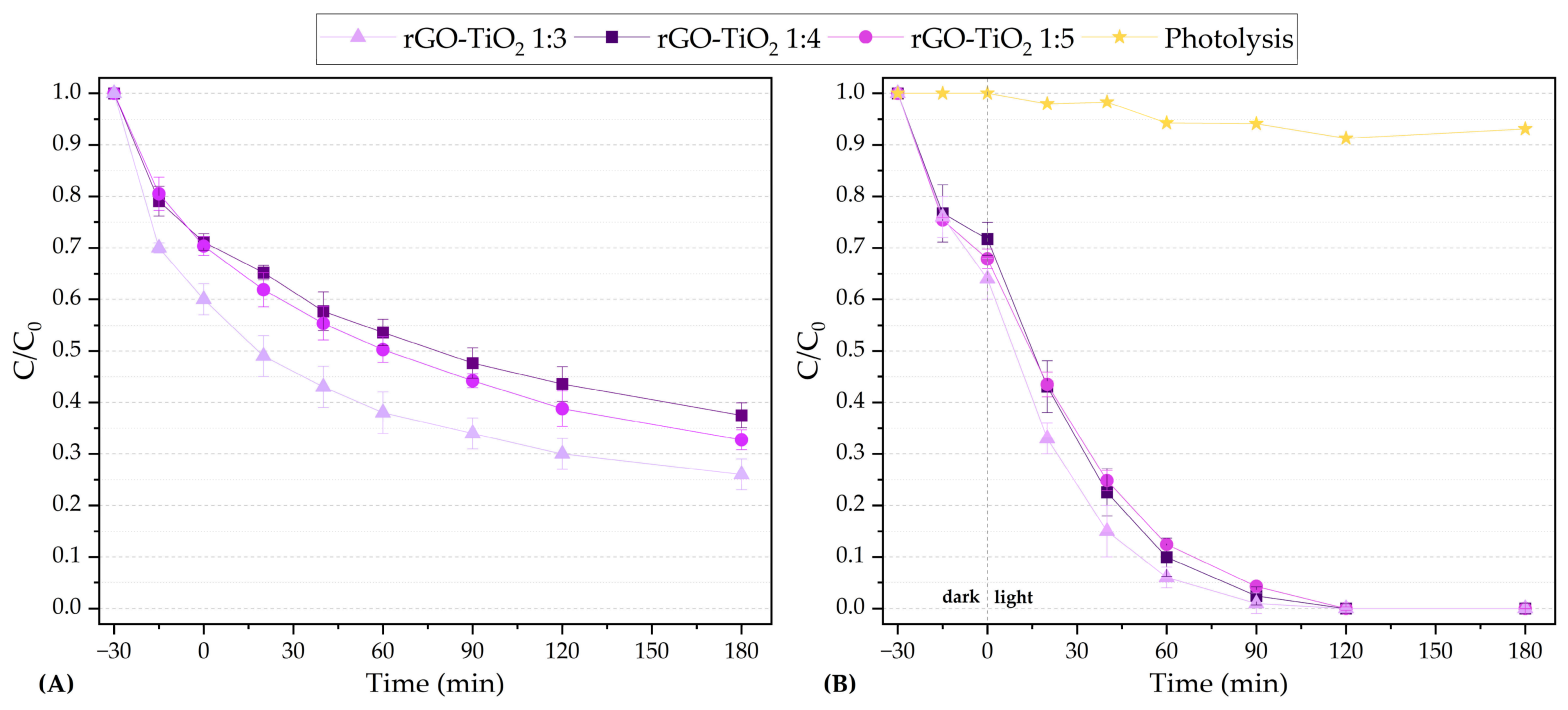
Normalized concentration of RhB solution during dark (**A**) and light (**B**) tests for samples with increased rGO:TiO_2_ ratio.

The effect of increasing TiO_2_ content was investigated by comparing the performance of rGO-TiO_2_ coatings with mass ratios of 1:4 and 1:5 to that of the standard 1:3 formulation (Figure 12). Under dark conditions (Figure 12A), higher TiO_2_ loadings did not result in improved performance, as demonstrated by the lower removal values of the 1:4 (63%) and 1:5 (67%) coatings, compared to the 74% removal achieved by the 1:3 formulation. Under UV-Vis irradiation (Figure 12B), all samples achieved complete RhB degradation within 2 h. After 90 min, the 1:3 formulation reached complete removal, whereas the 1:4 and 1:5 samples showed slightly lower values (98% and 96%, respectively). Therefore, increasing the TiO_2_ content does not result in faster or higher pollutant removal. This behavior may be attributed to TiO_2_ nanoparticle aggregation, which typically occurs at higher catalyst loadings and reduces the number of accessible active sites, thereby limiting both adsorption and photocatalytic activity. Similar observations have been reported in the literature, where excessive TiO_2_ contents led to nanoparticle clustering and decreased performance [43,110,111]. Evidence of aggregation is supported by optical microscopy and SEM images (Figure 7 and Figure S5), which show a less uniform coating at higher TiO_2_ contents, likely due to the less effective incorporation of TiO_2_ during the preparation of the coating dispersion, together with the possible loss of weakly bound nanoparticle agglomerates during the compressed air step used to eliminate excess coating.

As mentioned above, the overall decontamination performance can be attributed to the combined effects of adsorption and photodegradation. However, the specific mechanism involved in the photocatalytic process was not fully elucidated in this study. According to the literature, the photodegradation mechanism of rGO-TiO_2_ systems is based on the generation of reactive oxygen species, mainly ^•^OH and O_2_^•−^ radicals [112]. Due to the high specific surface area of rGO and the presence of oxygen-containing functional groups, the rGO-TiO_2_ system can adsorb pollutant molecules, thereby increasing their concentration at the photocatalytically active sites [113]. Upon irradiation, electron-hole pairs may be generated within TiO_2_. The photogenerated electrons can be transferred from the TiO_2_ conduction band to the rGO surface, where they may react with dissolved oxygen to generate O_2_^•−^ radicals. These species can subsequently participate in a series of reactions leading to the formation of ^•^OH radicals. Simultaneously, the photogenerated holes in the TiO_2_ valence band may oxidize H_2_O or OH^−^, also contributing to ^•^OH generation [30,66,114]. Therefore, adsorption and photodegradation are believed to act synergistically, with adsorbed molecules being degraded by the reactive species generated during irradiation [45].

Even though it was not directly demonstrated in the present work, the enhanced performance of rGO-TiO_2_ systems has often been associated with the ability of rGO to promote charge separation and suppress electron-hole recombination, as reported in several previous studies [110,115,116,117,118,119].

#### 2.2.1. Kinetics

Several studies have reported that the photocatalytic degradation kinetics of rGO-TiO_2_ systems can be described by a pseudo-first-order model [45,80,111,120]. Assuming a low dye concentration [45], the Langmuir–Hinshelwood model can be written according to Equation (1), where $C\left(t\right)$ is the dye concentration at a given time t, $C_{0}$ is the initial concentration, and $k_{app}$ is the kinetic constant of the whole process, accounting for the combined contribution of both adsorption and photocatalysis to the overall material performance. In this study, $C_{0}$ was considered as the concentration of the solution at t = 0 min, i.e., when the lamp was turned on.

(1) $$\ln\left[\frac{C\left(t\right)}{C_{0}}\right]=-k_{app}\cdot t$$

The analysis of the data obtained from different samples confirmed the validity of this assumption for rGO-TiO_2_-coated samples (Figure S6), yielding coefficients of determination (R^2^) higher than 0.98 (Table 6). Consistent with previous findings [80], the kinetic constant values of TiO_2_-containing coatings are one order of magnitude higher than those of rGO-coated samples and bare PU foams. In the case of bare PU samples, both untreated and 3 M NaOH pre-treated, as well as for rGO-coated samples, the lower k values indicate slower RhB removal kinetics. This behavior is consistent with the absence of TiO_2_ and suggests that adsorption represents the predominant contribution to the decontamination process. Regarding pre-treated foams, the slower decontamination efficiency is confirmed by the lower kinetic constant with respect to the untreated case (0.0446 1/min for rGO-TiO_2_ 1:3 and 0.0379 1/min for 3 M NaOH rGO-TiO_2_ 1:3). This might be explained by considering the increased stability of 3 M NaOH pre-treated foams and the consequently reduced detachment of coating fragments. In fact, in the case of untreated samples, detached fragments may create new surfaces with a higher area-to-volume ratio, providing additional sites for pollutant uptake and degradation, thereby contributing to the overall process efficiency.

For two- and three-layer rGO-TiO_2_ 1:3 coatings, the k values are lower than that of the monolayer case, suggesting that the presence of multiple layers may hinder contaminant diffusion and light penetration toward the active sites, thereby reducing the overall RhB removal efficiency. The increase in the TiO_2_:rGO mass ratio also resulted in slower decontamination performance, as confirmed by the kinetic constants of 1:4- (0.0381 1/min) and 1:5-coated samples (0.0310 1/min) with respect to the 1:3 formulation (0.0446 1/min).

#### 2.2.2. RhB Release Tests

To evaluate the potential RhB release from the used foams after the water decontamination tests, rGO and rGO-TiO_2_ samples were immersed in deionized water for three days. The results for untreated monolayer rGO- and rGO-TiO_2_ 1:3-coated samples have already been reported in [80].

**Figure 13:**
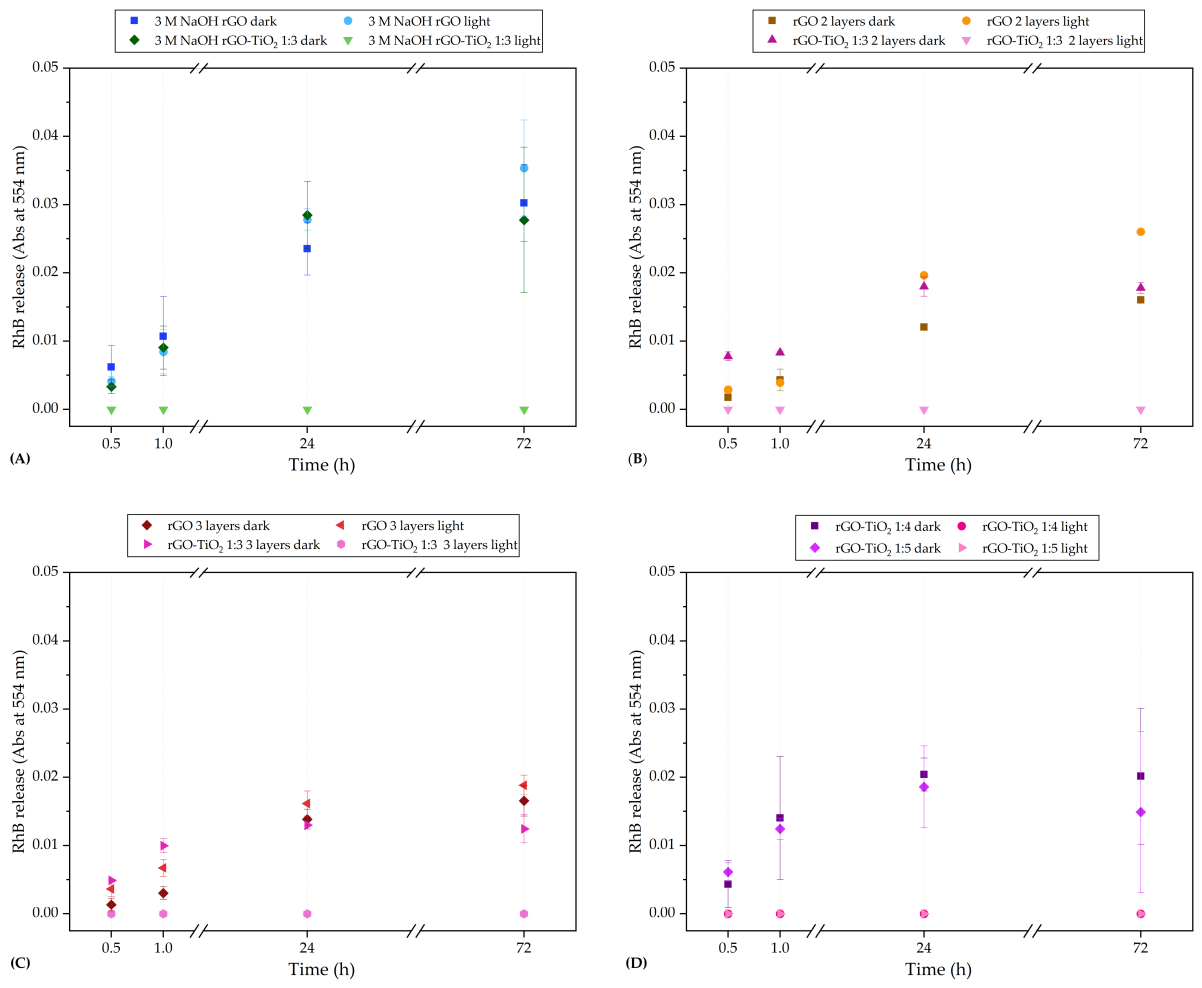
RhB release test results, expressed as water absorbance at 554 nm as a function of time, for (**A**) 3 M NaOH pre-treated rGO- and rGO-TiO_2_ 1:3-coated foams, (**B**) two-layer rGO- and rGO-TiO_2_ 1:3-coated foams, (**C**) three-layer rGO- and rGO-TiO_2_ 1:3-coated foams, and (**D**) rGO-TiO_2_ 1:4- and 1:5-coated foams, after water decontamination test.

As reported in Figure 13, a slight RhB release was observed for all rGO-coated foams, reaching a maximum after 72 h for both dark and irradiation tests. Despite this, the amount of released dye (maximum absorbance around 0.04) remained significantly lower than that of the initial 3 mg/L RhB solution (absorbance of 0.75 [80]). A similar trend was observed for all rGO-TiO_2_-coated samples used under dark conditions, which exhibited an absorbance peak after 24 h, followed by a slight decrease after 72 h. This behavior can be associated with a partial re-adsorption of RhB. In contrast, rGO-TiO_2_-coated samples exposed to UV-Vis light during the decontamination tests did not exhibit any detectable RhB release even after 72 h, further confirming the effective photodegradation capability of the TiO_2_-containing coatings.

## 3. Materials and Methods

### 3.1. Materials Preparation

Floating foams were prepared following the procedure described in [80]. Briefly, a GO commercial aqueous dispersion (4 mg/mL, Graphenea, San Sebastián, Spain) was chemically reduced with L-ascorbic acid (L-AA, Sigma Aldrich, St. Louis, MO, USA) for 24 h at room temperature, under stirring. A mass ratio of 1:10 between GO and L-AA was selected. Titanium dioxide nanopowder (TiO_2_, Degussa P25, Sigma Aldrich) was introduced during the last 30 min of the reduction period, considering GO:TiO_2_ mass ratios of 1:3, 1:4, and 1:5, to investigate the effect of TiO_2_ content on photocatalytic performance and coating resistance. A dispersion containing only rGO, without TiO_2_ addition, was also prepared for reference.

A commercial PU foam (20 pores per inch, 0.5 cm thickness), purchased from Modulor (Berlin, Germany), was manually cut into cylinders having a diameter of 5.5 cm. The obtained samples, without any preliminary treatment unless specified, were dip-coated with the rGO and rGO-TiO_2_ dispersions using a custom-made dip coater. Both the immersion and extraction velocities were set at 2.5 mm/s, and the immersion time was 30 s. Once extracted, the excess material was removed with compressed air (1–3 bar), and the samples were then oven-dried at 40 °C for 1 h. To remove the excess of L-AA, each foam was immersed four times in 300 mL of deionized water for 5 min in static conditions. Finally, the samples were oven-dried at 40 °C for 1 h. Foams were weighed initially and after the washing step to determine the amount of deposited material. Each weight measurement is reported as an average value with the corresponding standard deviation.

### 3.2. Foams Pre-Treatment

The samples were immersed in a 3 M NaOH solution [121] and subjected either to ultrasonic cleaning (Labsonic LBS1-6, Falc Instruments, Treviglio, Italy), with the addition of ice in the ultrasonic bath to prevent excessive solution overheating, or to mild stirring for different periods of time. With the aim of reducing the environmental and economic impact of the treatment, the reuse of the same 3 M NaOH solution up to five times was also assessed (Table 7). The initial concentration of the NaOH solution and the concentration after five use cycles were determined by acid-base titration, using a 1 M HCl solution as the titrant.

After treatment, the samples were rinsed with deionized water, following the procedure reported in Section 3.1, and subsequently dried in an oven at 40 °C for 1 h. The mass of each sample was measured before and after the pre-treatment to estimate the degree of hydrolysis (H), calculated according to Equation (2) [85].

(2) $$H(\%)=\frac{m_{start}-m_{pt}}{m_{start}}$$

where $m_{start}$ is the starting mass of the foam and $m_{pt}$ is the mass of the foam after the alkaline pre-treatment.

Subsequently, the pre-treated foams were coated using the previously described procedure.

### 3.3. Multilayer Coating

Multilayer coatings were prepared employing the rGO-TiO_2_ 1:3 formulation, as well as the rGO-only one, used as a control. In particular, the procedure described in Section 3.1, i.e., dip-coating, blowing with compressed air, and oven-drying at 40 °C for 1 h, was carried out two or three times on the same PU foam, yielding samples with two or three coating layers. Washing and final drying were then performed as previously described. The mass increase following each deposition, $∆m_{ij}$, was estimated as the difference between the weight of the sample after the current deposition, $m_{i}$, and that after the previous coating step, $m_{j}$ (Equation (3)).

(3) $$∆m_{ij}=m_{i}-m_{j}$$

To facilitate the comparison among the different experimental conditions investigated in this work, Table 8 summarizes the features of the prepared samples, including substrate pre-treatment, number of coating layers, and GO:TiO_2_ mass ratio. The samples are organized into different categories according to the parameter under investigation.

### 3.4. Materials Characterization

#### 3.4.1. Characterization Techniques

Bare and coated foams were observed with an optical microscope (Olympus sz-40, Hachioji-shi, Japan) at 10× magnification.

SEM-EDX analyses were performed with a Zeiss EVO 50 EP scanning electron (Oberkochen, Germany) microscope equipped with an Oxford INCA energy 2000 spectrometer (Oxfordshire, UK). Measurements were performed under high vacuum conditions (about 10^−4^ Pa), with an electron high tension (EHT) voltage of about 20 kV and a current probe of 150 pA. Images were acquired using both secondary and backscattered electrons at 100× magnification or different, when specified. Semi-quantitative EDX analyses were conducted on three independent areas at the same magnification. Before the analysis, the samples were sputtered with gold.

TGA-DTG analyses were conducted using a Discovery TGA 550 equipment (TA Instruments, New Castle, DE, USA) in a nitrogen atmosphere (40 mL/min flow rate), with a heating rate of 10 °C/min from room temperature to 800 °C.

DSC was carried out with a Mettler-Toledo DSC 823e instrument (Greifensee, Switzerland) over a temperature range between −60 °C and 200 °C. The protocol involved three ramps (heating/cooling/heating), with both heating and cooling rates set at 20 °C/min. The thermal transitions of PU and their corresponding temperature were determined from the analysis of the third ramp.

#### 3.4.2. Coating Stability Test

The stability of the final coating was evaluated by ultrasonic testing, subjecting the samples to theoretically more severe conditions than those used in the water decontamination tests, with the goal of quantifying material loss ($CL_{US}$, Equation (4)). Coated samples were immersed in 100 mL of deionized water and subjected to ultrasonication for 30 min in an ultrasonic bath (Labsonic LBS1-6, Falc Instruments). After treatment, the samples were oven-dried at 40 °C for 1 h and weighed.

(4) $$CL_{US}\left(\%\right)=\frac{m_{final}-m_{US}}{m_{final}-m_{start}}$$

where $m_{final}$ represents the foam dry mass after the washing cycle, $m_{US}$ is the foam mass after the stability test and $m_{start}$ is the starting mass of the foam. Although this test does not reproduce the actual operating conditions, it represents an accelerated and more severe stress scenario, providing valuable indications of coating stability.

#### 3.4.3. Water Decontamination Tests

The photocatalytic activity was assessed by evaluating the photodegradation of Rhodamine B (RhB) as a model pollutant. Each foam, contacted with 100 mL of a 3 mg/L RhB solution in the presence of mild magnetic stirring (250 rpm), was first placed in the dark for 30 min. Then, it was irradiated for 3 h with an Osram Ultra Vitalux UV-Vis lamp (Munich, Germany) placed 14 cm above the solution free surface and, consequently, above the floating foam. At this distance, the lamp emitted approximately 1200 W/m^2^ over its entire spectrum (280–2000 nm), including 35 W/m^2^ in the UVA region and 10 W/m^2^ in the UVB region, as measured with an HD2302.0 light meter (Delta OHM, Caselle di Selvazzano, Italy). To minimize temperature variations caused by lamp irradiation, the RhB solution was placed in a water-jacketed vessel with continuous water circulation, thereby maintaining constant thermal conditions throughout the experiment.

The degradation performance was monitored by analyzing periodic solution aliquots by UV-vis spectroscopy (Cary 5000, Jasco, Tokyo, Japan) at the characteristic absorption peak of RhB (554 nm). The photolytic degradation of RhB as well as adsorption experiments in the dark were performed with the same setup. All the experiments were performed in duplicate, and the results are reported as average values with the corresponding standard deviations. In the case of pre-treated samples, a coating loss parameter ($CL_{wt}$) was evaluated (Equation (5)) to quantify the percentage of coating lost during the water treatment.

(5) $$CL_{wt}\left(\%\right)=\frac{m_{final}-m_{wt}}{m_{final}-m_{start}}$$

where $m_{final}$ represents the foam dry mass after the washing cycle, $m_{wt}$ is the mass after the washing process, and $m_{start}$ is the initial foam mass.

The retention of RhB on the foams after decontamination tests was qualitatively assessed by visual inspection under UV light (366 nm).

Finally, RhB release tests were performed by immersing the used samples in 100 mL of deionized water for 72 h under static conditions, monitoring the solution absorbance at 554 nm by UV-vis spectroscopy.

## 4. Conclusions

This work focused on the development and characterization of 3D structured photocatalysts based on PU foams coated with a rGO-TiO_2_ nanocomposite coating, obtained through a simple and low-energy-consuming process. Different strategies have been investigated to improve coating adhesion or increase the amount of active material deposited. In the former case, an alkaline pre-treatment with 3 M NaOH proved effective in enhancing the stability of the deposited layers, reducing the loss of coating fragments that could represent an undesirable source of secondary pollution in the treated water. In the latter case, the obtainment of multilayer coatings and the increase in TiO_2_ content in the dispersion formulation resulted in an increase in the amount of active material; however, unlike the NaOH pre-treatment, these two approaches resulted in reduced integrity of the deposited layers.

The prepared PU foams were tested in simulated decontamination experiments using a 3 mg/L RhB solution, demonstrating good adsorption capacity and achieving complete photodegradation of the dye in 90 min for untreated PU coated with a single layer of the rGO-TiO_2_ 1:3 formulation. In contrast, NaOH pre-treated foams, as well as samples obtained with multilayer coatings or higher TiO_2_ loadings, exhibited a slightly slower decontamination process, requiring 120 min to achieve complete dye photodegradation. Nevertheless, the pre-treatment strategy appears promising, as it significantly reduces coating loss during operation, although further optimization is required to improve its effectiveness.

These results highlight the potential of the proposed materials for wastewater treatment, as they combine adsorption and photodegradation in the same system. At the same time, they underline the importance of achieving an optimal balance between photocatalytic activity and coating stability for future practical applications.

## Figures and Tables

**Table 1:** Average deposited coating mass and percentage of coating loss after ultrasonic test (CLus) for 3 M NaOH pre-treated foams coated with rGO.

Sample	Coating Mass (mg)	CLus (%)
rGO no pre-treatment	6.9 ± 0.7	31.7 ± 3.8
rGO US-300-60-R0	9.2 ± 0.9	19.2 ± 0.5
rGO US-100-60-R0	6.8 ± 0.1	39.0 ± 3.2
rGO US-100-60-R1	8.2 ± 0.5	37.2 ± 2.2
rGO US-100-30-R0	7.8 ± 0.9	34.1 ± 2.9
rGO US-100-30-R1	7.1 ± 0.4	35.5 ± 3.4
rGO US-100-30-R2	7.1 ± 0.2	30.0 ± 3.0
rGO US-100-30-R3	7.7 ± 0.9	20.5 ± 1.8
rGO US-100-30-R4	9.1 ± 2.4	23.6 ± 6.1
rGO ST-100-60-R0	9.7 ± 0.2	19.6 ± 6.6
rGO ST-100-120-R0	8.4 ± 2.3	25.3 ± 6.7
rGO ST-100-240-R0	7.6 ± 0.5	27.8 ± 1.4

**Table 2:** Average deposited coating mass and percentage of coating loss after ultrasonic test (CLus) for 3 M NaOH pre-treated foams coated with rGO-TiO_2_ 1:3.

Sample	Coating Mass (mg)	CLus (%)
1:3 no pre-treatment	18.6 ± 3.8	34.6 ± 4.1
1:3 US-300-60-R0	25.6 ± 1.4	10.6 ± 2.7
1:3 US-100-60-R0	25.3 ± 4.7	24.4 ± 0.6
1:3 US-100-60-R1	26.1 ± 2.3	21.9 ± 2.3
1:3 US-100-30-R0	27.1 ± 1.8	20.2 ± 5.0
1:3 US-100-30-R1	23.4 ± 0.7	26.0 ± 7.2
1:3 US-100-30-R2	22.1 ± 1.4	19.9 ± 4.7
1:3 US-100-30-R3	20.2 ± 5.3	20.6 ± 1.4
1:3 US-100-30-R4	19.0 ± 1.5	23.7 ± 2.9
1:3 ST-100-60-R0	21.9 ± 0.9	19.0 ± 3.9
1:3 ST-100-120-R0	27.2 ± 2.5	26.1 ± 4.5
1:3 ST-100-240-R0	19.7 ± 0.8	20.2 ± 1.1

**Table 3:** Average coating mass deposited after each deposition step (Δm), total coating mass measured at the end of the entire process, and percentage of coating loss after ultrasonic test (CLus) for rGO and rGO-TiO_2_ 1:3 coatings with 1, 2, or 3 layers.

Coating Type	$\Delta m_{1s}$ (mg)	$\Delta m_{21}$ (mg)	$\Delta m_{32}$ (mg)	Coating Mass (mg)	CLus (%)
rGO (1 layer)	80.2 ± 6.4	—	—	6.9 ± 0.7	31.7 ± 3.8
rGO (2 layers)	77.2 ± 6.6	27.3 ± 4.8	—	20.3 ± 6.0	42.0 ± 9.9
rGO (3 layers)	83.9 ± 3.9	28.4 ± 5.7	26.2 ± 8.3	25.6 ± 7.0	38.8 ± 2.1
rGO-TiO_2_ 1:3 (1 layer)	90.8 ± 5.9	—	—	18.6 ± 3.8	34.6 ± 4.1
rGO-TiO_2_ 1:3 (2 layers)	88.9 ± 4.8	62.3 ± 3.4	—	55.1 ± 2.5	42.7 ± 2.4
rGO-TiO_2_ 1:3 (3 layers)	92.7 ± 6.9	65.0 ± 3.9	56.6 ± 6.3	90.5 ± 4.8	47.8 ± 14.1

**Table 4:** Average deposited coating mass and percentage of coating loss after ultrasonic test (CLus) for rGO-TiO_2_ 1:3-, 1:4-, and 1:5-coated foams.

Coating Type	Coating Mass (mg)	CLus (%)
rGO-TiO_2_ 1:3	18.6 ± 3.8	34.6 ± 4.1
rGO-TiO_2_ 1:4	38.5 ± 2.8	44.2 ± 0.2
rGO-TiO_2_ 1:5	44.6 ± 1.7	54.6 ± 1.6

**Table 5:** Percentage of coating loss during water decontamination tests (CL_wt_) for untreated and 3 M NaOH pre-treated rGO- and rGO-TiO_2_ 1:3-coated foams.

	CL_wt_–Dark Test (%)	CL_wt_–Light Test (%)
rGO	31.0 ± 11.9	39.5 ± 3.1
rGO-TiO_2_ 1:3	12.9 ± 1.8	15.9 ± 1.1
3 M NaOH rGO	30.3 ± 2.1	31.3 ± 3.4
3 M NaOH rGO-TiO_2_ 1:3	10.5 ± 3.4	12.3 ± 2.1

**Table 6:** Pseudo-first-order kinetic constant (k) and coefficient of determination (R^2^) values obtained from RhB decontamination tests under UV-Vis irradiation.

Sample	k (1/min)	R^2^
PU	0.0048	0.9581
rGO	0.0044	0.9700
rGO-TiO_2_ 1:3	0.0446	0.9903
3 M NaOH PU	0.0046	0.9839
3 M NaOH rGO	0.0045	0.9883
3 M NaOH rGO-TiO_2_ 1:3	0.0376	0.9859
rGO 2 layers	0.0031	0.9950
rGO 3 layers	0.0043	0.9918
rGO-TiO_2_ 1:3 2 layers	0.0374	0.9728
rGO-TiO_2_ 1:3 3 layers	0.0349	0.9750
rGO-TiO_2_ 1:4	0.0381	0.9847
rGO-TiO_2_ 1:5	0.0310	0.9913

**Table 7:** Summary of the experimental conditions adopted for the foam pre-treatment, including soda volume, treatment time, number of reuse cycles of the NaOH solution, and treatment method.

	Soda Volume (mL)	Time (min)	Reuse Cycle	Treatment Method
US-300-60-R0	300	60	0	Ultrasounds
US-100-60-R0	100	60	0	Ultrasounds
US-100-60-R1			1	
US-100-30-R0	100	30	0	Ultrasounds
US-100-30-R1			1	
US-100-30-R2			2	
US-100-30-R3			3	
US-100-30-R4			4	
ST-100-60-R0	100	60	0	Stirring
ST-100-120-R0		120	0	
ST-100-240-R0		240	0	

**Table 8:** Classification and composition of the prepared samples, according to pre-treatment, number of deposited coating layers, and GO:TiO_2_ mass ratio.

Category	Sample Name	Pre-Treatment	Number of Layers	GO:TiO_2_ Mass Ratio ^b^
Reference	PU	No	—	—
	rGO	No	1	rGO only
	rGO-TiO_2_ 1:3	No	1	1:3
3 M NaOH pre-treatment	3 M NaOH PU	Yes ^a^	—	—
	3 M NaOH rGO	Yes ^a^	1	rGO only
	3 M NaOH rGO-TiO_2_ 1:3	Yes ^a^	1	1:3
Multilayer coating	rGO 2 layers	No	2	rGO only
	rGO-TiO_2_ 1:3 2 layers	No	2	1:3
	rGO 3 layers	No	3	rGO only
	rGO-TiO_2_ 1:3 3 layers	No	3	1:3
TiO_2_ loading increase	rGO-TiO_2_ 1:4	No	1	1:4
	rGO-TiO_2_ 1:5	No	1	1:5

^a^ PU pre-treatment was performed by immersing the foam in 300 mL of freshly prepared 3 M NaOH for 60 min subjected to ultrasonic treatment. This configuration corresponds to the code “US-300-60-R0” reported in Table 7, which was selected as the best candidate for subsequent investigations. ^b^ All coating dispersions were prepared by reducing a commercial GO dispersion with L-AA (GO:L-AA = 1:10 *w*/*w*) for 24 h. TiO_2_ was added during the last 30 min of the reduction time, as described in Section 3.1.

## Data Availability

The raw data supporting the conclusions of this article will be made available by the authors on request.

## Supplementary Materials

The following supporting information can be downloaded at https://www.mdpi.com/article/10.3390/molecules31152733/s1: Table S1. Initial foam mass and hydrolysis degree for all 3 M NaOH experimental conditions; Table S2. NaOH concentration determined by titration for the initial solution and after five use cycles; Table S3. Semi-quantitative EDS elemental composition of virgin and uncoated 3 M NaOH pre-treated polyurethane foams, expressed as normalized mass concentrations (%); Table S4. Thermal degradation parameters obtained from TGA/DTG analysis of pre-treated uncoated, rGO-coated and rGO-TiO_2_ 1:3-coated samples, including degradation temperature range, onset degradation temperature (T_onset_), maximum degradation temperature (T_max_), weight loss associated with each degradation stage, and residual mass at 800 °C; Table S5. Thermal degradation parameters obtained from TGA/DTG analysis of rGO-TiO_2_ 1:3, 1:4-, and 1:5-coated samples, including degradation temperature range, onset degradation temperature (T_onset_), maximum degradation temperature (T_max_), weight loss associated with each degradation stage, and residual mass at 800 °C; Figure S1. Surface details of 3 M NaOH pre-treated PU foams coated with rGO (A) and rGO-TiO_2_ 1:3 (B), obtained by SEM at 10,000× magnification, using secondary electrons; Figure S2. Third DSC heating ramp of (A) pre-treated bare PU and pre-treated rGO- and rGO-TiO_2_ 1:3-coated foams, (B) rGO-TiO_2_ 1:3-, 1:4-, and 1:5-coated foams; Figure S3. Cross sectional images of (I) rGO- and (II) rGO-TiO_2_ 1:3-coated 3 M NaOH pre-treated PU foams (A) before and (B) after stability test, obtained by SEM at 500× magnification using secondary electrons; Figure S4. Surface details of PU foams coated with 2 layers (A) and 3 layers (B) of rGO-TiO_2_ 1:3, obtained by SEM at 1000× and 2500× magnification, respectively, using secondary electrons; Figure S5. Surface details of rGO-TiO_2_ (A) 1:3-, (B) 1:4-, and (C) 1:5-coated foams obtained by SEM at (I) 500×, (II) 1000×, and (IV) 10,000× magnification, using secondary electrons. The corresponding Ti EDS elemental maps acquired at 1000× magnification are reported in (III); Figure S6. Pseudo-first-order kinetic modeling of RhB removal under UV-Vis light irradiation using (A) 3 M NaOH pre-treated foams (bare, rGO-, and rGO-TiO_2_ 1:3-coated foams); (B) foams coated with two and three layers of rGO and rGO-TiO_2_ 1:3; (C) rGO-TiO_2_ 1:4- and rGO-TiO_2_ 1:5-coated foams; (D) PU, rGO-, and rGO-TiO_2_ 1:3-coated foams.
