# Supplementary material for: Optimization of Reduced Graphene Oxide/Titanium Dioxide-Coated Polyurethane Foams as Novel Floating Photocatalysts for Water Decontamination
Source: Molecules. 2026 Aug 6;31(15):2733. doi: 10.3390/molecules31152733 (PMC13468509; doi:10.3390/molecules31152733)
Supplement: Supplementary file 1 [file molecules-31-02733-s001.zip › molecules-4398610-supplementary.pdf]

molecules

MDPI

Supplementary Materials

Optimization of Reduced Graphene Oxide/Titanium Dioxide-Coated Polyurethane Foams as Novel Floating Photocatalysts for Water Decontamination

Natalia Elia [1], Francesca De Rosa [1], Anna Dotti [1], Andrea Basso Peressut [1,*], Roberto Matarrese [2] and Saverio Latorrata [1,*]

1 Department of Chemistry, Materials and Chemical Engineering "Giulio Natta", Politecnico di Milano, Piazza Leonardo da Vinci 32, 20133 Milano, Italy; natalia.elia@polimi.it (N.E.); francesca.derosa@mail.polimi.it (F.D.R.); anna.dotti@polimi.it (A.D.)
2 Laboratory of Catalysis and Catalytic Processes, Department of Energy, Politecnico di Milano, Via La Masa 34, 20156 Milano, Italy; roberto.matarrese@polimi.it
* Correspondence: andreastefano.basso@polimi.it (A.B.P.); saverio.latorrata@polimi.it (S.L.)

Table S1. Initial foam mass and hydrolysis degree for all 3 M NaOH experimental conditions.

Sample	Initial Foam Mass (mg)	Hydrolysis Degree (%)
US-300-60-R0	334.8 ± 6.8	−0.08 ± 0.2
US-100-60-R0	329.5 ± 11.3	−0.03 ± 0.2
US-100-60-R1	341.3 ± 20.8	3.26 ± 5.4
US-100-30-R0	343.6 ± 6.7	0.28 ± 0.2
US-100-30-R1	339.6 ± 7.7	0.49 ± 0.1
US-100-30-R2	338.1 ± 2.8	0.27 ± 0.1
US-100-30-R3	332.9 ± 6.0	0.37 ± 0.1
US-100-30-R4	332.8 ± 3.0	0.26 ± 0.1
ST-100-60-R0	332.0 ± 2.8	0.36 ± 0.1
ST-100-120-R0	333.2 ± 5.7	0.18 ± 0.2
ST-100-240-R0	330.6 ± 3.3	0.27 ± 0.1

Table S2. NaOH concentration determined by titration for the initial solution and after five use cycles.

Sample	NaOH Concentration [mol/L]
Initial solution	3.02
Solution after 5 use cycles	2.70 ± 0.25

 https://doi.org/10.3390/molecules31152733

Table S3. Semi-quantitative EDS elemental composition of virgin and uncoated 3 M NaOH pre-treated polyurethane foams, expressed as normalized mass concentrations (%).

Sample	C	N	O
PU	63.58 ± 2.44	11.38 ± 1.47	25.03 ± 1.40
3 M NaOH PU	68.85 ± 3.18	10.39 ± 1.47	20.76 ± 3.51

Table S4. Thermal degradation parameters obtained from TGA/DTG analysis of pre-treated uncoated, rGO-coated, and rGO-TiO_2 1:3-coated samples, including degradation temperature range, onset degradation temperature (T_{onset}), maximum degradation temperature (T_{max}), weight loss associated with each degradation stage, and residual mass at 800 °C.

Sample	Degradation Range (°C)	T_{onset} (°C)	T_{max} (°C)	Weight Loss (%)	Residual Mass at 800 °C (%)
PU	200–450	~251	~299	~96	0
PU oven	200–300	~250	~283	~25	0
	300–450	~329	~359	~71	
3 M NaOH PU	200–300	~242	~271	~26	0.6
	300–450	~343	~363	~73	
rGO	200–300	~245	~281	~27	0
	300–450	~336	~326	~71	
3 M NaOH rGO	200–300	~245	~209	~26	1.7
	300–450	~353	~371	~71	
rGO-TiO_2 1:3	200–300	~247	~280	~23	3.7
	300–450	~333	~359	~68	
3 M NaOH rGO-TiO_2 1:3	200–300	~246	~280	~25	3.8
	300–450	~346	~364	~69	

Table S5. Thermal degradation parameters obtained from TGA/DTG analysis of rGO-TiO_2 1:3, 1:4-, and 1:5-coated samples, including degradation temperature range, onset degradation temperature (T_{onset}), maximum degradation temperature (T_{max}), weight loss associated with each degradation stage, and residual mass at 800 °C.

Sample	Degradation Range (°C)	T_{onset} (°C)	T_{max} (°C)	Weight Loss (%)	Residual Mass at 800 °C (%)
rGO-TiO_2 1:3	200–300	~247	~280	~23	3.7
	300–450	~333	~359	~68	
rGO-TiO_2 1:4	200–300	~247	~281	~23	6.3
	300–450	~344	~365	~68	
rGO-TiO_2 1:5	200–300	~250	~280	~24	7.1
	300–450	~344	~363	~68	

https://doi.org/10.3390/molecules31152733

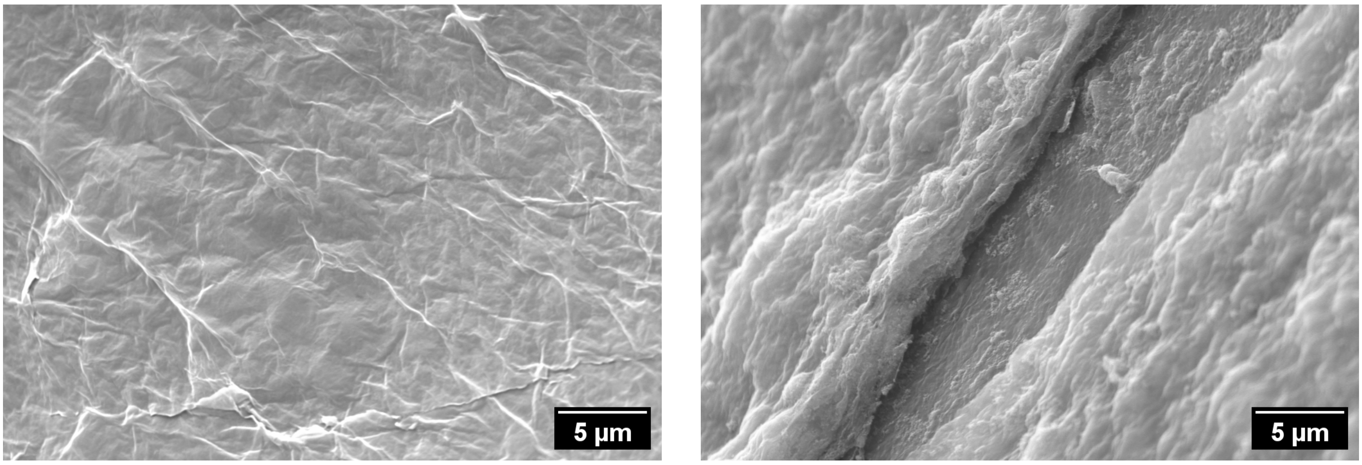

(**A**) (**B**)

Figure S1. Surface details of 3 M NaOH pre-treated PU foams coated with rGO (**A**) and rGO-TiO_2 1:3 (**B**), obtained by SEM at 10000× magnification, using secondary electrons.

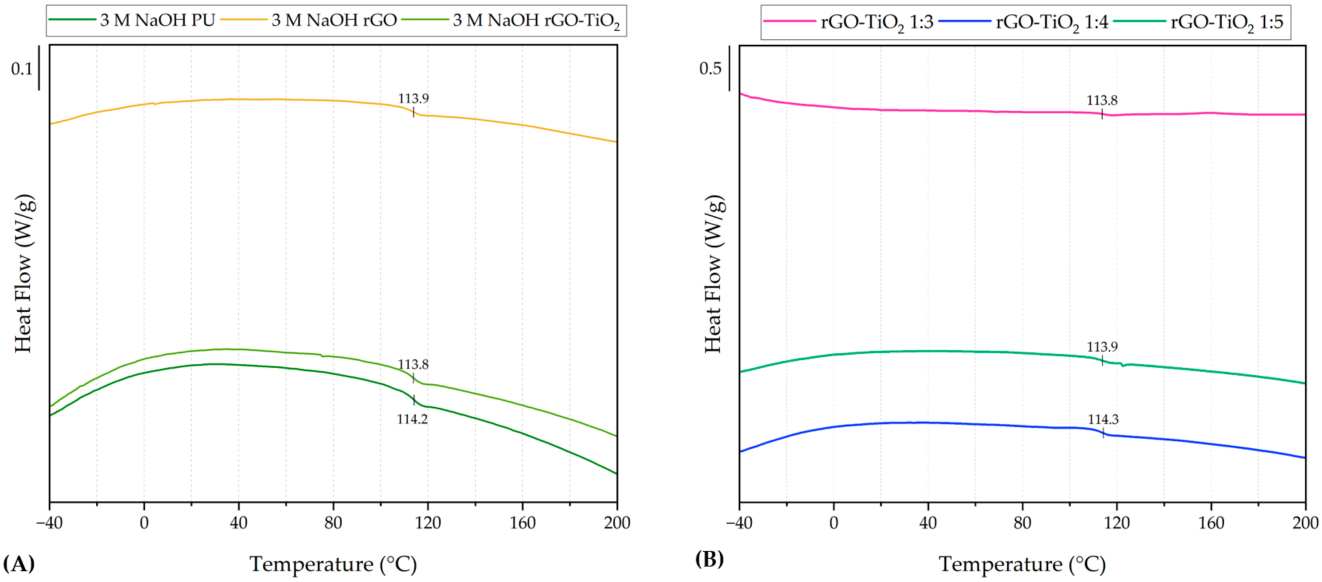

Figure S2. Third DSC heating ramp of (**A**) pre-treated bare PU and pre-treated rGO- and rGO-TiO_2 1:3-coated foams, (**B**) rGO-TiO_2 1:3-, 1:4-, and 1:5-coated foams.

(I)

(II)

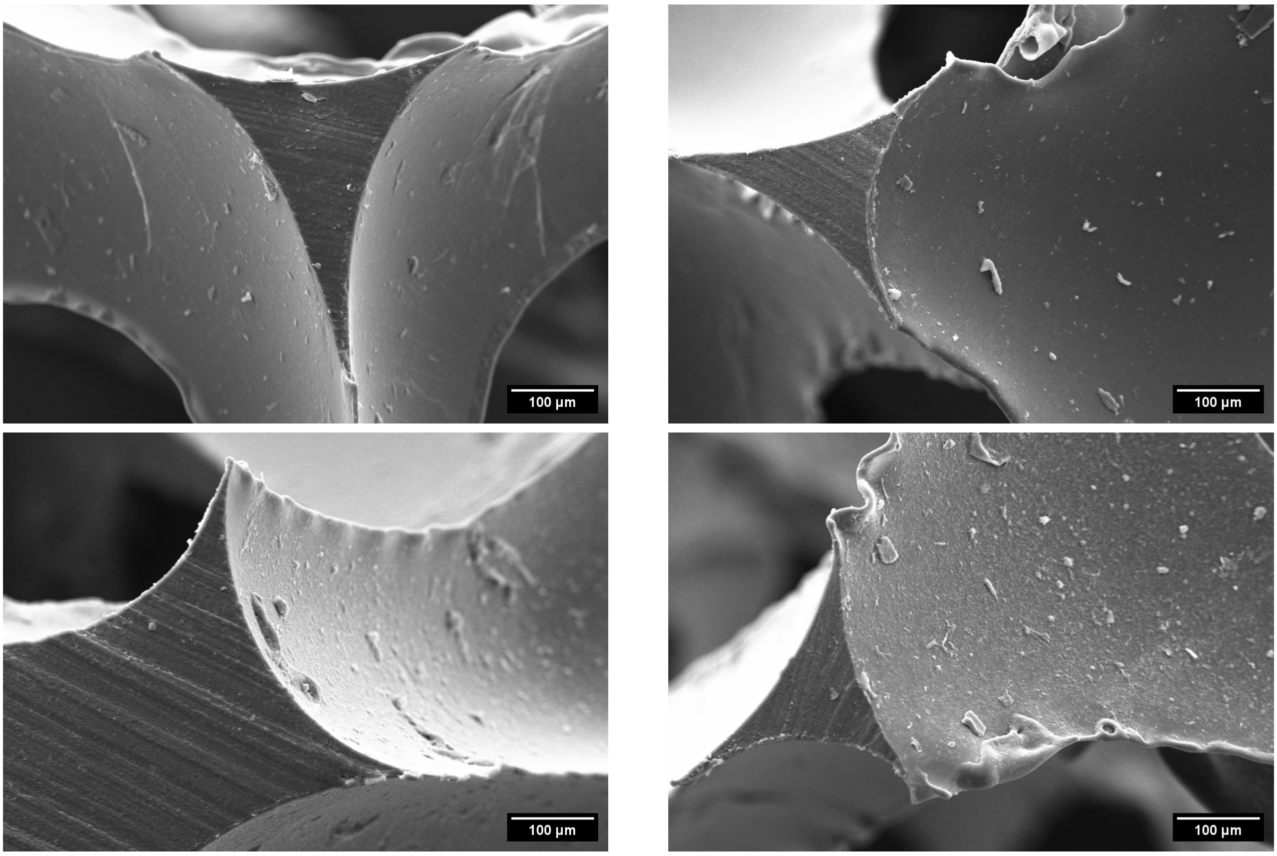

(**A**) (**B**)

Figure S3. Cross sectional images of (I) rGO- and (II) rGO-TiO_2 1:3-coated 3 M NaOH pre-treated PU foams (**A**) before and (**B**) after stability test, obtained by SEM at 500× magnification using secondary electrons.

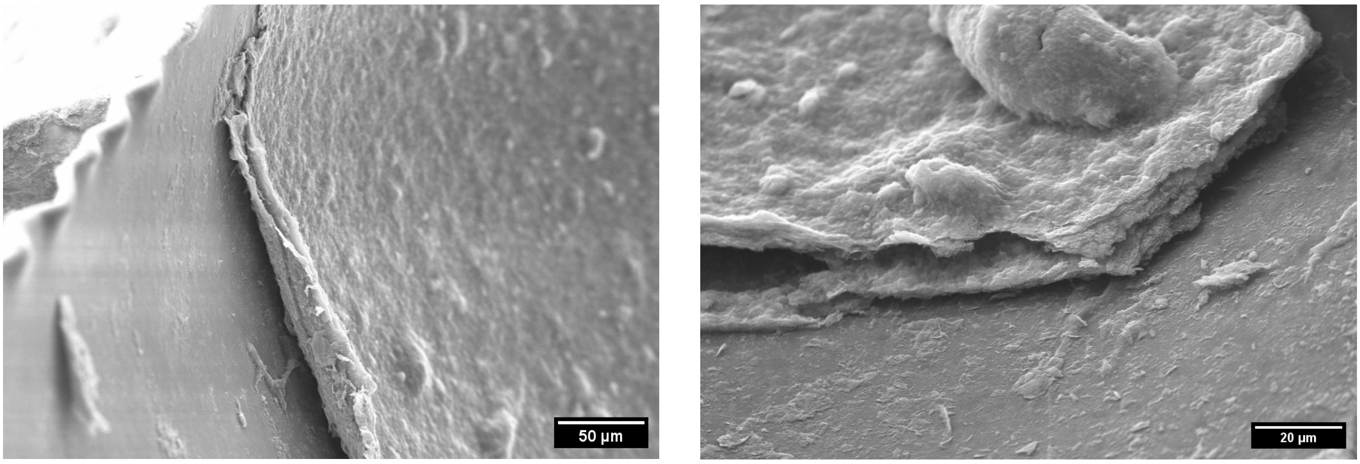

(**A**) (**B**)

Figure S4. Surface details of PU foams coated with 2 layers (**A**) and 3 layers (**B**) of rGO-TiO_2 1:3, obtained by SEM at 1000× and 2500× magnification, respectively, using secondary electrons.

https://doi.org/10.3390/molecules31152733

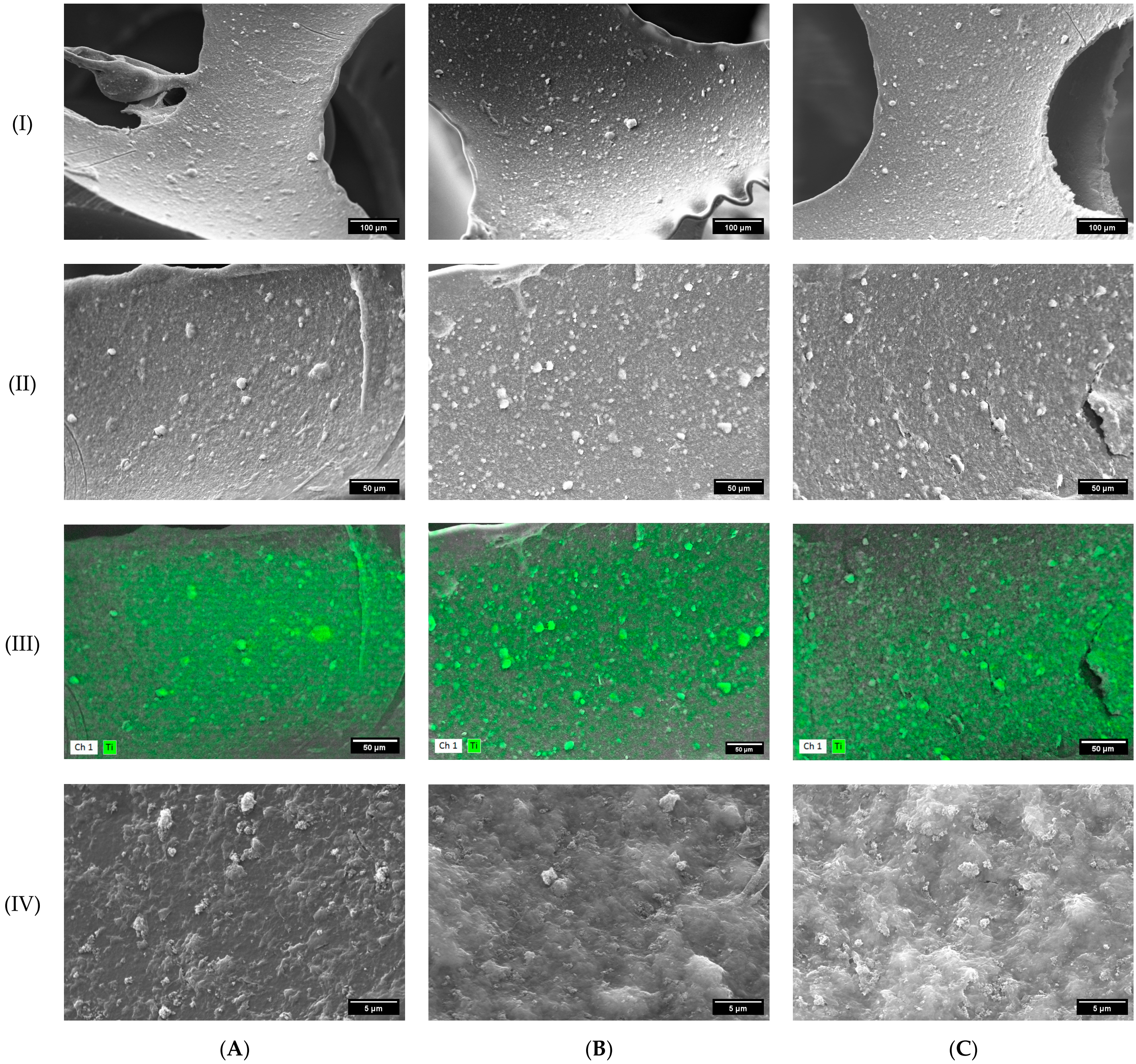

Figure S5. Surface details of rGO-TiO_2 (**A**) 1:3-, (**B**) 1:4-, and (**C**) 1:5-coated foams obtained by SEM at (I) 500×, (II) 1000×, and (IV) 10000× magnification, using secondary electrons. The corresponding Ti EDS elemental maps acquired at 1000× magnification are reported in (III).

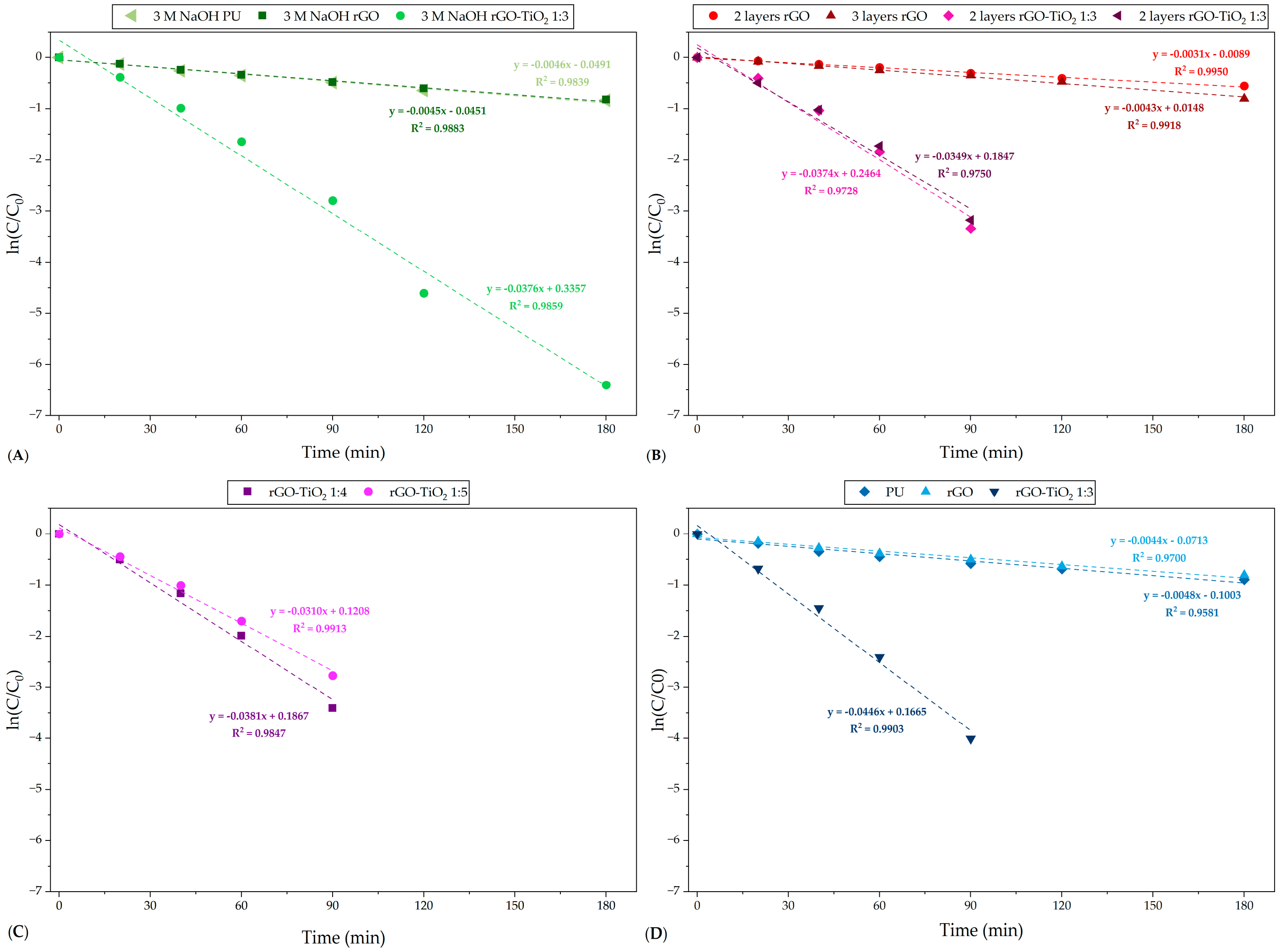

Figure S6. Pseudo-first-order kinetic modeling of RhB removal under UV-Vis light irradiation using (**A**) 3 M NaOH pre-treated foams (bare, rGO-, and rGO-TiO_2 1:3-coated foams); (**B**) foams coated with two and three layers of rGO and rGO-TiO_2 1:3; (**C**) rGO-TiO_2 1:4- and rGO-TiO_2 1:5-coated foams; (**D**) PU, rGO-, and rGO-TiO_2 1:3-coated foams.

https://doi.org/10.3390/molecules31152733
